# Piezoelectric materials for surface modification of titanium-based implants: material design, biological mechanisms, and translational challenges

**DOI:** 10.3389/fbioe.2026.1955725

**Published:** 2026-09-29

**Authors:** Qingshuo Zhan, Sirui Liu, Siyu Xie, Jiangqi Hu, Qian Pang, Bin Luo, Qingsong Jiang

**Affiliations:** Department of Prosthodontics, Beijing Stomatological Hospital, Capital Medical University, Beijing, China

**Keywords:** antibacterial activity, osseointegration, osteoimmune microenvironment, piezoelectric materials, surface modification, titanium-based implants

## Abstract

Titanium and its alloys are widely used in orthopedic and dental implants and bone repair scaffolds because of their mechanical properties, corrosion resistance, and biocompatibility. However, their bioinert surfaces may still show poor early osseointegration, bacterial infection, inflammatory imbalance, and limited long-term interfacial stability. Conventional surface modifications mainly change topography and chemistry, but provide limited simulation of the bone electrophysiological microenvironment. Piezoelectric materials convert physiological loading or external ultrasound into local electrical signals, offering a route to dynamically responsive titanium interfaces. This review summarizes piezoelectric material types, coating design, preparation methods, and biological effects on titanium surfaces. It discusses mechanisms related to osteogenic differentiation, osseointegration, antibacterial effects, infection control, and osteoimmune regulation. Piezoelectric coatings may affect membrane potential, ion channels, the cytoskeleton, cell adhesion, reactive oxygen species production, and macrophage function. Key translational challenges include signal decay in wet conditions, weak interfacial bonding, limited long-term stability, material safety concerns, lack of standardized preparation, and insufficient *in vivo* evaluation. Future studies should establish stable and biologically effective local electrical microenvironments and validate long-term safety in clinically relevant models.

## 1 Introduction

Titanium and titanium alloys are widely used in orthopedic implants, dental implants, and scaffolds for bone defect repair. This is mainly due to their good mechanical properties, corrosion resistance, and biocompatibility (Zhao et al., 2023). However, titanium-based implants are relatively bioinert. In the complex *in vivo* environment, they may still show poor early osseointegration, bacterial adhesion, biofilm formation, local inflammatory imbalance, and limited long-term interfacial stability (Yuan et al., 2021).

Surface modification has become an important strategy for improving the osseointegration and long-term stability of titanium implants. It can control surface microstructure and nanostructure, chemical composition, roughness, and wettability without changing the overall mechanical properties of the titanium substrate. Current methods mainly include surface treatments such as sandblasting, acid etching, anodic oxidation, and laser treatment. They also include coating methods such as plasma spraying, sol-gel processing, hydrothermal synthesis, and self-assembly (Xue et al., 2020). Sandblasting and acid etching, anodic oxidation, and bioactive coatings have shown positive effects on cell adhesion, osteogenesis, and antibacterial activity (Gehrke et al., 2018). However, most current studies focus on passive control of surface morphology and chemical composition. These approaches largely rely on predefined topography, chemical composition, or static bioactivity and cannot adapt to changing mechanical conditions or healing stages. Drug- or antibacterial-ion-releasing systems may also be limited by release kinetics, local concentration, long-term stability, and potential cytotoxicity. Consequently, they may not simultaneously address early infection control and later osseointegration. Less attention has been paid to reproducing the electrophysiological microenvironment between implants and bone tissue or providing active biological stimulation. Therefore, functional titanium surfaces with good interfacial bonding, biological activity, and electrical response are an important direction for further improving the biological performance of titanium implants.

Natural bone is not simply a mechanical support structure. It is an electroactive composite tissue. The piezoelectricity of bone tissue was formally proposed in 1957 (Fukada and Yasuda, 1957). Collagen fibers in the bone matrix have a non-centrosymmetric structure. Under compression, tension, or shear, they can produce a piezoelectric response and convert mechanical loads into local electrical signals (Isaacson and Bloebaum, 2010). During daily activities such as walking, mechanical loading can generate local potentials of approximately 300 μV in bone, termed stress-generated potentials. These signals are spatially nonuniform, and their magnitude and polarity vary with strain direction and loading state (Shrestha et al., 2026). This mechanical-to-electrical conversion is closely related to metabolic activities such as growth, bone remodeling, and fracture healing (Khare et al., 2020). Physiological loads from walking, mastication, joint movement, and muscle traction may generate weak but continuous bioelectrical signals in bone. These signals can further affect cell membrane potential, ion channel opening, cytoskeletal remodeling, and osteogenic signaling pathways. Therefore, reproducing the piezoelectric microenvironment of natural bone provides an important biomimetic approach for titanium implant surface modification.

In recent years, piezoelectric biomaterials have received wide attention in bone tissue engineering and implant modification. Lead-free inorganic piezoelectric materials, including barium titanate (BaTiO_3_), barium calcium zirconate titanate (BCZT), barium strontium titanate (BST), and zinc oxide (ZnO) have been used to build electroactive bone repair interfaces (Wu et al., 2020; Careta et al., 2021; Wu C. et al., 2023; Wu et al., 2024b). Organic piezoelectric polymers such as polyvinylidene fluoride (PVDF) and poly-L-lactic acid (PLLA), together with organic-inorganic composites, have also been studied (Liu et al., 2023; Fotoukian et al., 2024). Compared with conventional roughened surfaces, hydroxyapatite (HA) coatings, or antibacterial ion coatings, piezoelectric materials provide a dynamic response. They can generate local electrical potential or charge under physiological loading, low-intensity pulsed ultrasound (LIPUS), occlusal force, or cell traction. In this way, external mechanical stimulation is converted into electrical signals that cells can sense. However, most studies focus on single-material systems, one preparation method, or one biological function. Existing reviews have advanced piezoelectric biomaterials and implant-surface functionalization from several perspectives. However, few specifically address titanium-based piezoelectric coatings. The links among material composition, fabrication, coating structure, wet-state signal generation and transfer, cellular and tissue responses, and long-term stability remain insufficiently integrated.

Based on this background, this review focuses on the use of piezoelectric materials for surface modification of titanium-based implants. It discusses material selection, structural design, preparation methods, and biological effects of piezoelectric coatings on titanium surfaces. This article is a narrative review. Literature searches were conducted in Web of Science, PubMed, and Google Scholar, supplemented by reference tracking of key studies. Search terms were combined by topic, including materials, fabrication and polarization, interfacial engineering, osteogenesis, antibacterial activity, osteoimmune regulation, and long-term stability. Representative terms included “titanium implant,” “piezoelectric coating,” “fabrication,” “osteogenesis,” and “antibacterial.” Original studies directly addressing titanium-based piezoelectric coatings were prioritized, together with representative reviews and mechanistic studies from related fields. The review primarily covers literature published from 2000 to 2026 while retaining earlier foundational studies. First, the basic principles of the piezoelectric effect and the main types of piezoelectric biomaterials are introduced. Second, key factors in coating design are summarized, including interfacial bonding, surface topography, three-dimensional porous structures, mechanical activation methods, long-term stability, and biocompatibility. The main preparation methods are then reviewed. The mechanisms involved in osteogenic differentiation and osseointegration, antibacterial and anti-infective effects, and immune regulation are also discussed. Finally, current challenges and future directions are considered. This review aims to support the design and further translation of piezoelectric coatings on titanium-based implants from the perspective of interfacial electrical microenvironment regulation.

## 2 Piezoelectric biomaterials

### 2.1 Mechanism of the piezoelectric effect

The piezoelectric effect occurs when external mechanical force causes a relative displacement between positive and negative charge centers in a material. This displacement changes the electric dipole moment and produces surface charge or electrical potential. Based on the direction of energy conversion, the piezoelectric effect can be divided into direct and inverse effects. In the direct effect, compression, tension, bending, or shear produces an electrical potential. This process converts mechanical energy into electrical energy. In the inverse effect, an external electric field causes mechanical deformation (Figure 1). Piezoelectricity is closely related to a non-centrosymmetric material structure. In a centrosymmetric material, force-induced displacement of positive and negative charge centers often cancels out in space. Therefore, a clear macroscopic piezoelectric response is difficult to produce. In a non-centrosymmetric structure, force can produce an uneven charge distribution, and microscopic changes in dipole moments appear as surface charge accumulation (Chorsi et al., 2019). Different piezoelectric materials work through different mechanisms. In inorganic materials, the response mainly depends on changes in ionic positions within the crystal structure. For example, deformation of the lead zirconate titanate (PZT) or BaTiO_3_ lattice shifts the centers of positive and negative charges and forms an electric dipole moment (Ramadan et al., 2014). In organic materials, the response mainly depends on the orientation of molecular dipoles and the arrangement of polymer chains. In the β phase of PVDF, parallel molecular dipoles produce a strong piezoelectric response (Wan and Bowen, 2017). The piezoelectricity of PLLA is related to the orientation of C=O dipoles in its molecular chains (Oh et al., 2020). Crystal structure, polarization level, nanoscale orientation, pore structure, and interfacial charge also influence the piezoelectric effect (Liu et al., 2020). To avoid conceptual ambiguity, this review treats piezoelectricity, ferroelectricity, static polarization, and piezocatalysis as related but noninterchangeable phenomena. Piezoelectricity describes load-dependent dynamic electrical signals generated by mechanical deformation. Ferroelectricity refers to spontaneous polarization that can be oriented or switched. Static polarization primarily denotes residual surface charge or potential that persists without repeated mechanical loading. By contrast, piezocatalysis involves mechanically or ultrasonically induced charge separation and may generate reactive oxygen species or trigger drug or ion release. Therefore, biological outcomes should not be attributed solely to piezoelectricity because a system contains a piezoelectric material. Activation mode, static charge, topography, wettability, ion release, and catalytic reactions must also be considered.

**FIGURE 1:**
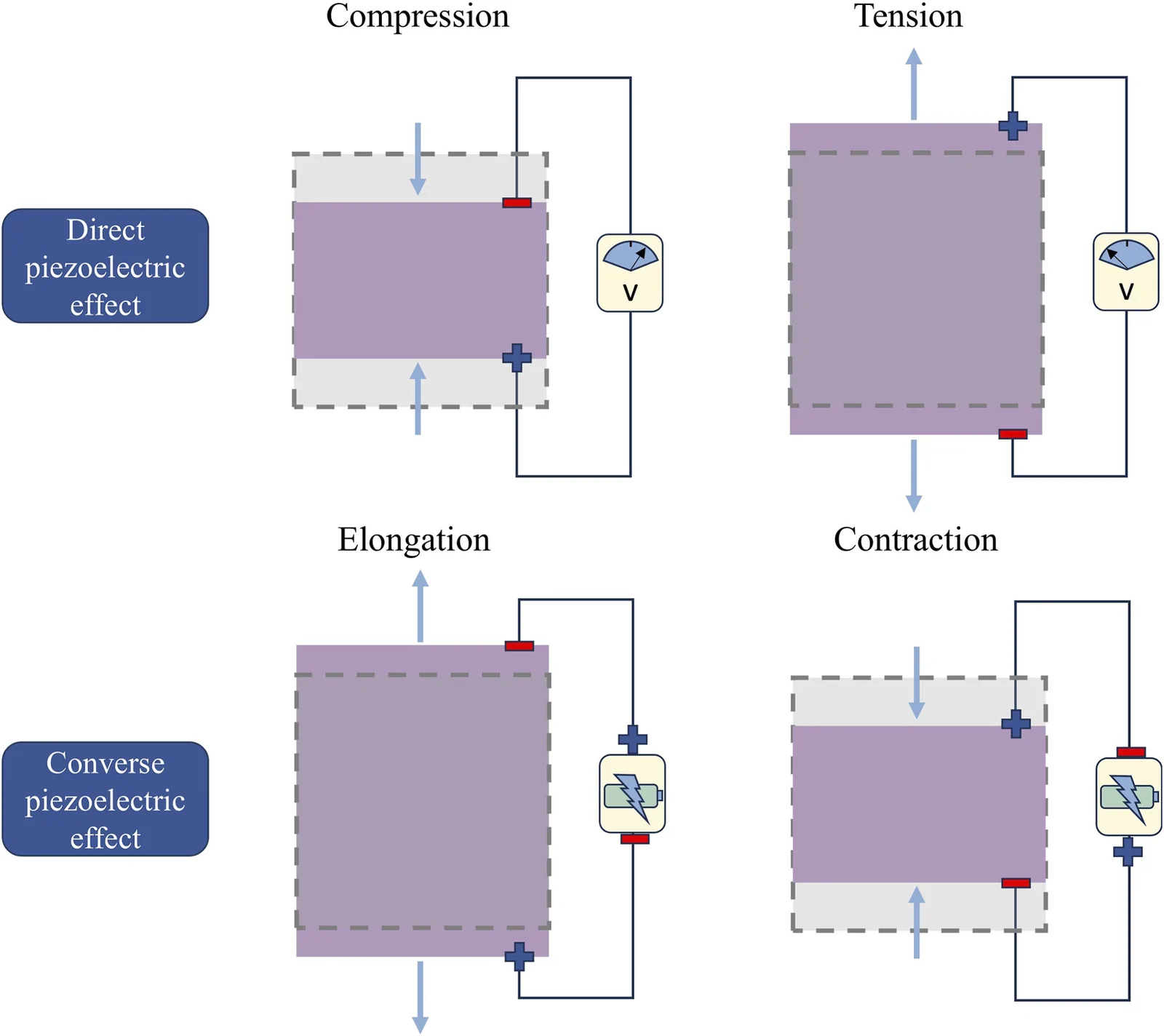
Schematic illustration of the direct and converse piezoelectric effects in piezoelectric materials.

### 2.2 Classification

#### 2.2.1 Inorganic piezoelectric materials

Inorganic piezoelectric materials usually have high piezoelectric coefficients, stable crystal structures, and strong electromechanical conversion. They are the most widely used materials in conventional piezoelectric devices. Their piezoelectricity mainly arises from stress-induced displacement between positive and negative charge centers in non-centrosymmetric crystals. In ferroelectric ceramics, an external field can align ferroelectric domains, producing macroscopic polarization and enhancing the piezoelectric response. Representative materials include PZT, barium titanate BaTiO_3_, and ZnO.

PZT is a typical perovskite ferroelectric ceramic formed by a solid solution of PbZrO_3_ and PbTiO_3_. Under mechanical stress, Ti^4+^ or Zr^4+^ shifts off-center relative to surrounding ions. This displacement separates charge centers, changes the unit-cell dipole moment, and generates a piezoelectric response (Zhao et al., 2025). Its strong piezoelectricity is closely associated with compositions and crystal structures near the morphotropic phase boundary (MPB). Nevertheless, the high Pb content of PZT, which can exceed 60 wt%, raises substantial environmental and long-term biosafety concerns (Aksel and Jones, 2010). These concerns have motivated intensive efforts to develop lead-free piezoelectric alternatives.

BaTiO_3_ is a typical lead-free perovskite ferroelectric ceramic whose behavior is closely linked to temperature-dependent structural transitions. Above its Curie temperature, approximately 120 °C, BaTiO_3_ adopts a centrosymmetric cubic paraelectric phase without spontaneous polarization. Near room temperature, structural symmetry breaking separates the charge centers and produces spontaneous polarization, enabling ferroelectric and piezoelectric responses. BaTiO_3_ has a high dielectric constant and good ferroelectric properties, while avoiding the potential safety concerns associated with Pb in PZT (Huang et al., 2025). Its electrical response is strongly structure-dependent and varies with phase, grain size, microstructure, composition, defect state, and processing history (Jiang et al., 2019).

Beyond perovskite ferroelectric ceramics, inorganic materials with other crystal structures can also be piezoelectric. ZnO is a representative nonferroelectric piezoelectric material with a non-centrosymmetric wurtzite structure. Mechanical stress deforms its lattice and displaces positive and negative charge centers, generating piezoelectric polarization. Reported coefficients are approximately 3–20 pC/N for d_33_ and −5 pC/N for d_31_ (Cafarelli et al., 2021). The response is also scale-dependent because nanoscale surface defects and lattice contraction or expansion can alter piezoelectricity (Zhao et al., 2004).

#### 2.2.2 Organic piezoelectric materials

Synthetic organic piezoelectric materials mainly include PVDF, poly (vinylidene fluoride-co-trifluoroethylene) [P(VDF-TrFE)], and PLLA. PVDF and P(VDF-TrFE) are the most widely studied synthetic piezoelectric polymers. PVDF has several crystal phases, including the α, β, γ, and δ phases. The α phase has little net dipole moment and therefore shows weak piezoelectricity. The β phase adopts an all-trans chain conformation, and its ordered molecular dipoles provide the structural basis for the strong PVDF piezoelectric response. Accordingly, β-phase content, crystallinity, and the orientation of polymer chains and dipoles are major determinants of PVDF piezoelectricity (Szewczyk et al., 2020; Jin et al., 2021).

P(VDF-TrFE) is a ferroelectric copolymer of vinylidene fluoride (VDF) and trifluoroethylene (TrFE). Steric effects introduced by TrFE favor and stabilize the ferroelectric β phase at room temperature (Gallucci and Hunt, 2026). The intrinsic longitudinal coefficient d_33_ of PVDF and P(VDF-TrFE) is typically approximately −30 pC/N, reflecting a negative longitudinal response unlike that of most inorganic ceramics (Liu and Wang, 2020).

PLLA is another representative synthetic organic piezoelectric material. Its piezoelectricity mainly comes from the ordered orientation of C=O dipoles in ester groups. Unlike PVDF, the α phase of PLLA is piezoelectrically active and does not require additional poling. Under mechanical loading, the number and ordered arrangement of C=O dipoles along PLLA chains determine its response.

Natural piezoelectric materials include collagen, cellulose, chitosan, silk fibroin, gelatin, and demineralized bone matrix (Wang et al., 2022). These materials come from biological systems and usually have good flexibility, biocompatibility, and biodegradability (Shin et al., 2020). However, their piezoelectric responses are weaker than those of piezoelectric ceramics and some synthetic polymers. Their electrical properties are also strongly influenced by molecular or crystal orientation, domain arrangement, and assembled structure (Wang et al., 2020). Collagen is a representative natural piezoelectric protein. Its response is associated with dipole accumulation along peptide chains and hydrogen bonding within supramolecular assemblies (Lay et al., 2021). Type I collagen fibers show a pronounced shear response, with an effective coefficient of approximately 2 pm/V for a single fiber (Minary-Jolandan and Yu, 2009). In natural materials, incomplete macromolecular ordering, amorphous regions, and structural heterogeneity can partially cancel local polarization and limit the macroscopic response. Native silk fiber bundles have a reported response of approximately 1 pC/N (Fukada, 1956), which can be enhanced by increasing molecular orientation and controlling crystallinity (Sencadas et al., 2019). Natural polymers such as polysaccharides and proteins often serve as flexible and biocompatible matrices in piezoelectric composites. They can be combined with inorganic fillers such as BaTiO_3_ and ZnO to improve the overall electrical response (Zhang et al., 2018; Sriplai et al., 2020).

#### 2.2.3 Composite piezoelectric materials

Piezoelectric composites have become an important strategy for combining the advantages of inorganic and organic materials while reducing their individual limitations. By adding inorganic piezoelectric particles to an organic polymer matrix, these composites can provide relatively high piezoelectric output, flexibility, and biocompatibility. For example, adding BaTiO_3_ and ZnO to PVDF can improve piezoelectric performance when the nanoparticle content is properly controlled (Al-Ejji et al., 2025). For piezoelectric modification of titanium implant surfaces, composite coatings may work together with surface morphology and bioactive components. They may therefore promote osseointegration, reduce bacterial adhesion, and improve long-term implant stability. This combination can improve key properties such as mechanical performance, piezoelectric response, and biocompatibility (Cho et al., 2025).

## 3 Design of piezoelectric materials for titanium surface modification

The long-term function of a piezoelectric coating on a titanium substrate in the complex *in vivo* environment does not depend only on the piezoelectric properties of the material. Coating design is equally important. Piezoelectric performance, interfacial bonding, surface morphology, electrical signal transfer, biocompatibility, and long-term stability should all be considered.

### 3.1 Piezoelectric performance

The biological function of a piezoelectric coating on a titanium surface first depends on whether it can produce a stable and effective response. Piezoelectric performance is determined not only by the material type. It is also influenced by polarization state, microstructure, coating thickness, and interfacial structure. Polarization treatment is an important step for improving macroscopic piezoelectric performance. Suitable electric field strength, temperature, and treatment time can align ferroelectric domains or molecular dipoles. This alignment increases remanent polarization and piezoelectric output. However, an excessively high electric field or unsuitable treatment conditions may cause breakdown, structural damage, or unstable polarization. Coating thickness is also important. An overly thin coating may be affected by substrate clamping, lattice mismatch strain, interfacial dead layers, and depolarization field screening. These effects can reduce the effective piezoelectric coefficient and electromechanical conversion (Kelley et al., 2020). Wang et al. found that increasing the thickness of an aluminum nitride (AlN) piezoelectric layer significantly enhanced the ultrasonic echo response. Electrode thickness and multilayer structure also affected the total output by improving density and interfacial bonding (Wang et al., 2024). Increasing PZT film thickness also increased remanent polarization (Pr) and the effective longitudinal piezoelectric coefficient (d_33_) (Nguyen et al., 2013). However, a thicker coating may cause residual stress, cracking, and weaker interfacial bonding. Overall, no unified relationship has yet been established between coating thickness, piezoelectric output, and biological performance. Piezoelectric coatings should be optimized by balancing thickness, interfacial stability, and functional output.

For titanium-based piezoelectric coatings, mechanical loading is transferred from the titanium substrate to the piezoelectric layer across the coating–substrate interface, producing strain and generating load-dependent polarization charges and local surface potentials. The effective electromechanical response of the coating depends not only on the intrinsic piezoelectric coefficient of the material but also on interfacial bonding, substrate constraint, coating thickness, and elastic compatibility between the constituent layers (Ali et al., 2004). Studies of PZT thin films, for example, have shown that elastic constraints imposed by substrate clamping and local substrate deformation can alter the measured piezoelectric response (Nguyen et al., 2017). The deformation-induced polarization charge is concentrated primarily at the coating surface, thereby establishing a local potential at the piezoelectric coating–fluid interface. In physiological fluids, counterions accumulate near the charged surface and form an electrical double layer, which partially screens the interfacial electric field and causes the potential to decay rapidly with distance from the surface. Piezoelectric activity under wet conditions is therefore better understood as the dynamic modulation of the local electrical potential and ionic environment near the coating, rather than as an unattenuated macroscopic electric field acting directly on distant cells (Bortel et al., 2026). Several parallel pathways may couple this near-surface electrical environment to biological responses. Changes in surface potential and local ion distribution may alter the amount and conformation of adsorbed proteins and the accessibility of their cell-binding domains, which may in turn influence integrin-mediated cell adhesion, spreading, and downstream signaling (Tang et al., 2018; Metwally and Stachewicz, 2019; Wan et al., 2021). Dynamic surface potentials may also alter the cell membrane potential and may thereby facilitate the opening of voltage-gated Ca^2+^ channels, Ca^2+^ influx, and activate Ca^2+^-dependent osteogenic signaling pathways (Zheng et al., 2025). Surface charge may further regulate the interfacial adsorption and local distribution of Ca^2+^ and phosphate ions, potentially creating favorable conditions for calcium phosphate deposition and hydroxyapatite nucleation (Wu C. et al., 2023; 2024b). In materials with piezocatalytic activity, mechanically induced charge separation may drive interfacial redox reactions and generate reactive species that contribute to antibacterial activity and immune modulation (Sun et al., 2024; Ding et al., 2026). Piezoelectric stimulation may also indirectly affect osteogenesis and angiogenesis by modulating macrophage inflammatory signaling and paracrine activity (Wu H. et al., 2023; Zheng et al., 2025). However, these pathways have largely been supported separately in different materials and experimental systems and should not be interpreted as a single causal cascade that has been validated from end to end within the same titanium-based piezoelectric coating system. Furthermore, dry-state output and intrinsic d_33_ should not be treated as direct measures of *in vivo* stimulation. Table 1 summarizes the available quantitative data reported for representative titanium-based piezoelectric coatings. Notably, these values were obtained from different material systems, loading conditions, and test environments. They are therefore descriptive rather than prescriptive and do not constitute recommended design targets. The inconsistent testing protocols, together with the frequent absence of adhesion and wet-state electrical-output data, currently preclude the definition of universal performance thresholds.

**TABLE 1:** Quantitative comparison of representative piezoelectric coatings on titanium surfaces.

Material	Coating architecture	Fabrication method	Activation condition	Piezoelectric/electrical performance	Coating thickness and adhesion	Ref.
BaTiO_3_	BaTiO_3_ ceramic coating on a porous Ti6Al4V scaffold	Electron beam melting (EBM) of porous Ti6Al4V followed by wet-chemical BaTiO_3_ coating	LIPUS: 1.5 MHz, 200 μs, 1 kHz, and 30 mW cm^-2^; 20 min daily *in vivo*	d_33_ and electrical output were not directly reported	Thickness and adhesion strength: Not reported	(Fan et al., 2020)
Ba_1-x_Sr_x_TiO_3_ (BST)	BST rod array on Ti, with a rod diameter of 329.5 ± 4.41 nm and length of 1709.9 ± 15.27 nm	Two-step hydrothermal conversion of a TiO_2_ rod array, followed by polarization	LIPUS was evaluated	d_33_ = 320 p.m. V^−1^; polarization induced orientation-dependent local surface potentials	Thickness and adhesion strength: Not reported	(Wu et al., 2024b)
Ba_0_._85_Ca_0_._15_Zr_0_._1_Ti_0_._9_O_3_ (BCZT)	Integrated BCZT/TiO_2_ conversion coating derived from TiO_2_ nanotubes	Anodization to form TiO_2_ nanotubes, followed by hydrothermal conversion at 240 °C for 6 h and polarization	Physiological loading or LIPUS	Residual polarization charge and loading-induced piezoelectric charge were reported; d_33_: Not reported	Thickness: ∼26.8 μm; adhesion strength: Not reported	(Wu et al., 2023a)
PLLA/HA	PDA-anchored electrospun PLLA/HA fibrous coating containing 5–20 wt% HA	PDA priming layer on Ti followed by electrospinning of PLLA/HA fibers	Mechanical loading at 5 Hz; force magnitude: Not reported	Piezoelectric sensitivity: 7.41 ± 0.096 mV N^−1^ for neat PLLA; 2.41 ± 0.192, 4.41 ± 0.316, 4.577 ± 0.423, and 5.42 ± 0.192 mV N^−1^ for 5, 10, 15, and 20 wt% HA, respectively	Thickness: Not reported; PDA increased peel adhesion, but the numerical value was not explicitly stated	(Fotoukian et al., 2024)
PWH/GelMA/SA	Dual-network GelMA/SA hydrogel containing piezoelectric whitlockite (PWH) nanoparticles on Ti	Alkali-heat treatment and PDA modification of Ti, followed by formation of a GelMA/SA dual-network hydrogel containing PWH; poled at 5 kV cm^-1^ for 30 min	LIPUS: 1.0 MHz, 0.6 W cm^-2^, 50% duty cycle, 60 s d^-1^	d_33_ = 4.8 ± 0.5 pC N^−1^ for Ti-GS/3PWH and 6.1 ± 1.4 pC N^−1^ for Ti-GS/5PWH	Thickness: Not reported; PDA improved adhesion, and no obvious delamination was observed after 14 days of LIPUS exposure	(Wang et al., 2026)
BaTiO_3_	BaTiO_3_ coating on a gyroid-sheet TPMS porous Ti alloy scaffold	EBM fabrication of the TPMS Ti6Al4V scaffold, BaTiO_3_ coating, and corona polarization	dynamic cyclic loading	d_33_ and surface zeta potential were measured; the BT/pG group showed a higher d_33_ than the BT/pF group, but numerical values were not explicitly stated	Thickness and adhesion strength: Not reported	(Wu et al., 2025)
TiO_2_@BaTiO_3_–vancomycin	Vancomycin-loaded TiO_2_@BaTiO_3_ coaxial nanotube coating	Anodization to form TiO_2_ nanotubes, hydrothermal formation of the BaTiO_3_ coaxial layer, drug loading, and polarization	no external mechanical activation	Residual polarization charge produced a dam-like drug-retention effect; d_33_ and dynamic output: Not reported	Thickness and adhesion strength: Not reported	(Wu et al., 2021c)
BST-Ag	Ag nanoparticle-decorated BST rod array on Ti alloy	Hydrothermal formation of BST rods followed by photochemical Ag deposition and polarization	no external mechanical activation reported	d_33_ = 1.22 pC N^−1^ for BST-Ag1.0; residual negative charge promoted local Ag^+^ retention	Thickness and adhesion strength: Not reported	(Wu et al., 2024a)
3DMA (TiO_2_/PVDF)	Three-dimensional multifunctional architecture comprising a lower TiO_2_ nanotube layer and an upper PVDF piezoelectric nanofiber layer	Anodization to form TiO_2_ nanotubes followed by direct electrospinning of PVDF nanofibers	Fiber bending–release and cell–fiber mechanical interactions	Peak power density of the piezoelectric nanogenerator: 3.57 nW cm^-2^	Total sample thickness: ∼0.2 mm; interfacial adhesion: Not reported	(Sun et al., 2023)
PVDF	PVDF film on laser-textured Ti6Al4V	Laser texturing followed by PVDF deposition	static electroactivity and hydrophobicity were evaluated	d_33_, piezoelectric voltage, and current output: Not reported	Thickness and adhesion strength: Not reported	(Silva et al., 2026)
BaTiO_3_-x/L-arginine	Oxygen-deficient BaTiO_3_-x nanorod array on Ti, grafted with L-arginine	Hydrothermal conversion to BaTiO_3_, NaBH_4_ reduction to generate oxygen vacancies, and L-arginine grafting	Ultrasound: 1.0 MHz, 1.5 W cm^-2^, 50% duty cycle, 15 min	Ultrasound-induced piezoelectric current was measured; maximum PFM amplitude: ∼140 pm; d_33_: Not reported	Nanostructure was reported to be stably bonded to the substrate; thickness and quantitative adhesion strength: Not reported	(Sun et al., 2024)
BaTiO_3_/Mn-MIL-100	BaTiO_3_ nanotubes decorated with Mn-MIL-100 nanoenzyme tips	Ti anodization at 80 V for 1 h, hydrothermal conversion to BaTiO_3_ nanotubes, and Mn-MIL-100 assembly	Ultrasound: 0.75 W cm^-2^ for 7 min	Effective d_33_: 28.53 p.m. V^−1^ for BNT and 66.51 p.m. V^−1^ for BNT/Mn-MIL-100; output voltage: ∼0.2 and ∼1 V, respectively	Thickness and adhesion strength: Not reported	(Ding et al., 2026)
BaTiO_3_	BaTiO_3_ particle coating on an EBM porous Ti6Al4V scaffold	EBM fabrication of porous Ti6Al4V, wet-chemical BaTiO_3_ coating, and corona polarization	LIPUS: 1.5 MHz, 0.2 m, 1 kHz, 30 mW cm^-2^, 10 min	d_33_ was measured; LIPUS-induced current was 10.2 μA *in vitro* and ∼6.7–6.8 μA in subcutaneous tissue	Mean pore size: ∼450 μm; coating thickness and adhesion strength: Not reported	(Wu et al., 2023b)
ferroTi (ferroelectric BaTiO_3_)	In situ-grown dense BaTiO_3_ nanostructured coating on Ti; nanoparticle diameter ∼100 nm	Sequential alkali-heat treatment, Ba^2+^ ion exchange, hydrothermal growth of BaTiO_3_, and corona polarization	1 MHz ultrasound at 0–2 W cm^-2^, typically 1 W cm^-2^ for 5 min in cell experiments	Static surface potential: 553.6 mV at 10 kV poling; dynamic piezoelectric potential: 750 mV at 2 W cm^-2^; d_33_ was measured	Thickness: ∼300 nm; adhesion strength: >30.20 ± 1.18 MPa	(Li et al., 2026)

### 3.2 Interfacial bonding strength

The bonding strength of a coating determines whether it can function during implantation, mechanical loading, and bone remodeling. It is therefore directly related to long-term implant survival. Bonding strength is affected by both the internal cohesion of the coating and adhesion at the coating-substrate interface. Related factors include coating composition, properties, structure, and the surface features of the titanium substrate. Piezoelectric ceramics and metallic titanium often differ in thermal expansion, elastic modulus, and chemical reactivity. Direct high-temperature sintering or deposition of a thick coating may form a brittle interfacial layer, pores, or residual stress, which can lead to delamination. Poon et al. prepared a BCZT/calcium titanate (CaTiO_3_) bilayer coating on Ti-6Al-4V. They found that reactions at the metal-ceramic interface produced a porous region and that moisture absorption by calcium oxide (CaO) affected coating adhesion (Poon et al., 2021). This finding shows that intermediate layers and heat treatment conditions are important for lead-free ceramic coatings. Common strategies for improving bonding strength include three approaches. First, titanium dioxide (TiO_2_) nanotubes, micro-arc oxidation layers, or hydroxyapatite layers can serve as transition layers. They increase substrate roughness and improve mechanical interlocking (Liu et al., 2023; Wen et al., 2023). Second, adhesive layers such as dopamine or polydopamine can strengthen interactions between organic coatings and titanium substrates (Huang et al., 2016). Third, piezoelectric particles can be embedded in porous titanium surfaces or the pore walls of three-dimensional scaffolds to improve mechanical locking (Fan et al., 2020). Transition layers, surface roughening, control of interfacial reactions, and mechanical interlocking can therefore improve bonding between piezoelectric coatings and titanium substrates.

### 3.3 Surface topography and microstructure

The surface microstructure and nanostructure of titanium affect not only cell adhesion and osseointegration, but also local amplification and transfer of piezoelectric signals. A rough and porous surface can increase hydrophilicity and provide a larger area for protein adsorption and ion exchange. Nanotubes, nanosheets, nanoparticles, and fibrous structures can influence cytoskeletal tension. They can therefore work with electrical stimulation to regulate cell behavior. A study of ZnO nanosheet coatings showed that nanosheet arrays produced a stronger local electric field than dense films under small strains caused by cell activity. They also promoted Saos-2 cell proliferation and the expression of early osteogenic markers (Careta et al., 2021).

### 3.4 Design of three-dimensional porous structures

Three-dimensional printed porous titanium scaffolds are widely studied for large bone defects and reconstruction of vertebral bodies or femoral head necrosis (Gu et al., 2022). Their pore size, porosity, and elastic modulus can be adjusted to improve mechanical behavior and bone ingrowth (Song et al., 2023). However, porous titanium itself is not electroactive and cannot actively regulate the electrical microenvironment. A piezoelectric coating can address this limitation. Fan et al. coated porous Ti-6Al-4V scaffolds with BaTiO_3_ piezoelectric ceramic. The coating provided electroactivity while maintaining the original pore size, porosity, and strut structure of the scaffolds (Fan et al., 2020).

### 3.5 Mechanical activation methods

The structural design of a piezoelectric coating should first consider the mechanical environment at the implantation site. Piezoelectric materials require mechanical stimulation to achieve effective mechanical-to-electrical conversion. The source, direction, frequency, and intensity of loading should therefore guide coating design for different implant sites. For load-bearing orthopedic implants, daily activities such as walking, running, and jumping generate cyclic bone strain. *In vivo* measurements show that tibial strain increases markedly with activity intensity. Peak axial tensile strain during running and vertical jumping is approximately 1.94-fold and 3.92-fold that during walking, respectively. In addition, peak axial compressive strain in the distal tibia is approximately 1.95-fold that at the midshaft (Milgrom et al., 2022). These findings indicate that bone strain is strongly dependent on both activity level and anatomical location. Piezoelectric coatings on orthopedic implants should therefore accommodate the dynamic strain conditions associated with different activities and implantation sites to maintain stable mechanical-to-electrical conversion. For dental implants, occlusal force during mastication is the primary physiological mechanical load. *In vivo* measurements indicate that implant loading varies with individual factors, chewing patterns, and prosthetic design. In some cantilever restorations, local implant loads can exceed 600 N during mastication (Assoratgoon et al., 2022). A load of approximately 100 N has been used to represent normal mastication, while loading away from the implant’s central axis produces greater bending moments and peri-implant bone stress and strain (Robinson et al., 2019). Both the magnitude and direction of masticatory loading vary dynamically and include substantial lateral components (Assoratgoon et al., 2022). Piezoelectric coatings on dental implants should therefore be designed to accommodate dynamic, multiaxial occlusal loads ranging from tens to hundreds of newtons, while maintaining effective mechanical-to-electrical conversion under both axial and lateral loading.

### 3.6 Long-term stability

Implants usually remain in the body for a long period. Piezoelectric coatings must maintain structural integrity and stable electrical function in the complex *in vivo* environment. Material classes can undergo different structural changes in simulated physiological environments. For degradable PLLA, phosphate-buffered-saline exposure can disrupt fibers, cause local acidification, and reduce β-phase content, suggesting progressive loss of piezoelectricity. A higher molecular weight may delay this process (Zhang et al., 2024). Saline immersion and cell culture can also release Ba and alter the surface composition of BaTiO_3_ coatings (Vandrovcova et al., 2021). However, corresponding changes in post-immersion output or polarization retention have rarely been measured. For inorganic ceramic coatings, important concerns include crystal phase stability, fatigue cracking, and possible ion release. For polymer coatings such as PVDF and PLLA, β phase loss, polarization decay, degradation rate, and changes after sterilization should be considered. Stability should not be evaluated only by initial characterization after preparation. Piezoelectric response, surface potential, and bonding strength should be tested again after simulated body fluid immersion, cyclic loading, ultrasound stimulation, or sterilization. Repeated loading may cause coating fatigue in load-bearing orthopedic and dental implants. Piezoelectric performance may also decrease over time.

### 3.7 Biocompatibility

Material selection for piezoelectric coatings on titanium surfaces must also consider biosafety. Lead-containing piezoelectric ceramics have excellent piezoelectric properties, but possible toxicity makes them unsuitable for long-term implants (Aksel and Jones, 2010). Even for lead-free materials, the release ranges of Ba^2+^, Sr^2+^, Zn^2+^, Cu^2+^, and Ag^+^ require attention. A suitable amount of ion release may promote osteogenesis, antibacterial activity, or immune regulation. Excessive release may cause cytotoxicity, inflammation, or local tissue damage (Zhang et al., 2024). Therefore, coating design should not simply add multiple functional components. The role and safe range of each component should be clearly defined. Safety should be assessed through release profiles, cytotoxicity, inflammatory factors, osteogenic differentiation, and *in vivo* tests.

## 4 Preparation methods for piezoelectric coatings

Fabrication is not merely the deposition of a piezoelectric material on titanium; it determines the structure and function of the interface. Substrate pretreatment, film formation, heat treatment, and poling can alter phase composition, crystallinity, orientation, polarization, thickness, porosity, morphology, and interfacial integrity. These changes subsequently affect electromechanical conversion, mechanical stability, and biological performance. Even for the same material, different processes can produce markedly different microstructures and piezoelectric responses. Fabrication should balance output, interfacial stability, and biological function through controlled structure–process relationships.

### 4.1 Hydrothermal synthesis and in situ conversion

Fabrication generally comprises substrate pretreatment, formation of the piezoelectric layer, and post-treatment. Pretreatment provides reaction sites and changes roughness, wettability, mechanical interlocking, and adhesion. For P(VDF-TrFE)/BaTiO_3_ coatings on Ti-30Ta, polished and acid-etched substrates showed critical adhesion strengths of approximately 9.56 and 8.36 MPa, respectively (Ribeiro et al., 2022). This difference indicates contributions from topography, surface energy, wettability, and mechanical interlocking. For titanate coatings, TiO_2_ nanotubes or porous TiO_2_ layers are commonly produced by anodization or micro-arc oxidation. Subsequent hydrothermal reactions with Ba^2+^, Ca^2+^, Zr^4+^, or Sr^2+^ form TiO_2_@BaTiO_3_, BCZT/TiO_2_, or BST interfaces (Wu et al., 2020; Wu et al., 2023 C.; 2024b). Hydrothermal conditions control phase formation, particle size, thickness, and nanoscale morphology. Prolonged secondary treatment can transform BaTiO_3_ nanostructures from spherical to cubic morphologies and promote the cubic-to-tetragonal transition. Effective coefficients of approximately 10, 60, and 180 pm/V have been reported under different conditions, accompanied by distinct cellular and osteogenic responses (Zheng et al., 2023). These methods offer good bonding because TiO_2_ provides reaction sites and mechanical interlocking. However, time, temperature, and precursor composition must balance target-phase formation, structural retention, thickness, and uniformity.

### 4.2 Electrospinning

Electrospinning simultaneously forms fibrous films and regulates electroactive phases in polymers such as PVDF, making it important for flexible interfaces (Xue et al., 2019). It creates an extracellular-matrix-like network on titanium and promotes chain orientation during electric-field drawing. Solution composition, voltage, flow rate, and tip-to-collector distance jointly affect fiber diameter, crystallinity, β-phase content, and output. These variables are coupled, so a universal linear relation between any single parameter and piezoelectricity is unlikely (Yin et al., 2022). Adding HA, ZnO, or other bioactive fillers can enhance piezoelectric response, osteoconductivity, and mineralization (Fotoukian et al., 2024; Vaidya et al., 2025). Electrospinning is mild and strongly biomimetic, but membrane thickness, pore stability, long-term wear, and post-sterilization performance require systematic evaluation.

### 4.3 Physical thin-film deposition

Physical thin-film deposition produces dense piezoelectric layers with uniform composition and controlled thickness while regulating crystal structure and orientation. For sputtered PZT films, target composition, substrate, temperature, atmosphere, and annealing affect composition, crystallinity, phase ratio, preferred orientation, and grain size. Substrate lattice matching can change the proportions of (001) (110), and (111) orientations under otherwise similar growth conditions. Film thickness can also directly regulate effective response. In PZT systems, d_33_ initially increases with thickness and then approaches a plateau. This trend may reflect reduced residual stress, a smaller relative contribution from low-performance interfacial layers, and grain growth (Ma et al., 2021). Dense sputtered ZnO films are uniform, whereas high-area hydrothermal nanosheets more readily generate local stimulation under cell-induced microstrain (Careta et al., 2021). Accordingly, fabrication of piezoelectric thin films should involve the coordinated control of film thickness, crystallographic orientation, grain size, residual stress, and substrate-clamping effects, as these parameters collectively govern the effective piezoelectric response of the coating. Overall, physical deposition can form stable material layers that do not easily break down. It also causes little change to the original substrate morphology. However, it provides limited coverage of inner walls in complex three-dimensional porous scaffolds and requires expensive equipment. Differences in thermal expansion and elastic modulus between the coating and substrate may also weaken interfacial bonding and limit further use (Xue et al., 2020).

### 4.4 Electrophoretic deposition

Electrophoretic deposition (EPD) forms particulate or composite coatings on conductive substrates using relatively simple equipment and can accommodate complex geometries. For example, BaTiO_3_, HA, or other bioactive ceramic particles can be deposited on Ti-6Al-4V by electrophoretic deposition. The resulting composite coating can provide both piezoelectric response and osteoconductivity (Jaafar et al., 2022; Nair and Viannie, 2024). Suspension composition, voltage, deposition time, and heat treatment determine coating structure and performance. At 10 V for 10 min, varying the BaTiO_3_/HA ratio produced coatings approximately 10–60 μm thick. Increasing BaTiO_3_ from 40 to 60 wt% increased tetragonality and Vickers hardness, while composite d_33_ increased from 145 to 159 pC/N. These findings indicate that the composition of the deposition system can further modulate the mechanical and piezoelectric properties by altering the crystal structure and relative proportion of ceramic phases (Nair and Viannie, 2024). However, d_33_ was not measured directly on the titanium-supported coating. Quantitative links among EPD parameters, coating microstructure, and actual output remain unclear. Overall, EPD allows convenient adjustment of particle loading and composite composition. However, post-deposition drying and sintering may further affect coating densification, residual stress, and interfacial bonding. Therefore, its processing parameters require further optimization to balance piezoelectric performance with long-term stability.

### 4.5 Layer-by-layer assembly

Layer-by-layer (LbL) assembly enables secondary functionalization of piezoelectric interfaces through the alternate deposition of different functional components. By varying the number and composition of deposition layers, it can regulate surface morphology and the spatial distribution of functional species. Current studies have mainly used LbL to construct functional outer layers on pre-existing piezoelectric materials. For example, sequential assembly of negatively charged antibacterial nanoparticles, cationic polyelectrolytes, and antibiofilm enzymes on a β-PVDF piezoelectric film increased surface roughness. The degree of roughness also varied with nanoparticle type and the number of deposition cycles, indicating that LbL assembly can regulate the surface microstructure of piezoelectric interfaces. Importantly, increasing the number of LbL layers did not markedly weaken the piezoelectric effect of the underlying PVDF layer. This finding suggests that surface functionalization and the electromechanical conversion of the underlying layer can coexist to some extent. Biologically, LbL functional layers can provide antibacterial and antibiofilm activity during the early stage when mechanical stimulation is absent. Under dynamic mechanical stimulation, the piezoelectric response of the underlying PVDF layer can promote preosteoblast proliferation. This arrangement provides complementary functions, namely, early infection control followed by dynamic electrical stimulation (Carvalho et al., 2024). Therefore, LbL assembly is better suited to secondary functionalization of piezoelectric interfaces, including the integration of antibacterial, bioactive, or drug-delivery functions. However, its application in titanium-based piezoelectric implants remains limited. The long-term stability of interlayer bonding under physiological-fluid immersion and cyclic mechanical loading also requires further verification.

### 4.6 Poling treatment

Poling is another major determinant of final coating performance. In materials with ferroelectric domains or orientable dipoles, poling promotes directional alignment and strengthens the macroscopic response. Poling conditions can also alter surface charge and interfacial properties. For a PVDF coating on TiO_2_-nanotube-modified titanium, poling at 85 °C for 30 min produced different d_33_ values as voltage increased. At d_33_ = 2.61 pC/N, the contact angle decreased from approximately 108° in the unpoled state to 47° (Wu et al., 2021b). In HA/PVDF coatings, increasing d_33_ to approximately 1.52 pC/N reduced the contact angle to 31.7° and enhanced *in vitro* mineralization (Wu et al., 2021b). Surface charge and hydrophilicity decay during storage, whereas physiological cyclic loading can regenerate charge and delay this loss (Wu et al., 2021a). Thus, poling not only establishes the initial piezoelectric state of a material, but the poling parameters and long-term stability of the polarized state also influence surface charge, wettability, and mineralization of the coating. Dynamic piezoelectric responses induced by mechanical loading may help maintain these interfacial properties. Achieving spatially uniform poling of piezoelectric coatings on complex three-dimensional porous titanium scaffolds, however, remains an unresolved processing challenge.

### 4.7 Sterilization considerations

Sterilization is essential before implantation, but it may also alter material surface properties. Steam autoclaving, gamma irradiation, ultraviolet irradiation, and plasma treatment can each affect the chemical composition, wettability, and roughness of titanium surfaces to different extents, thereby modifying cellular responses (Yin et al., 2022). These effects require particular attention for titanium surfaces with micro- or nanoscale features. For example, steam autoclaving can damage the TiO_2_ nanoporous structure produced by anodization, whereas ultraviolet and gamma irradiation have relatively limited effects on surface morphology (Guo et al., 2021). For piezoelectric materials, sterilization must also preserve electromechanical function. Electrospun P(VDF-TrFE) fibers subjected to 70% ethanol cleaning and overnight ultraviolet exposure retained an average peak voltage of approximately ±0.4 V under mechanical loading of approximately 8 mN at 2–3 Hz. Neither the intensity nor the quality of the piezoelectric signal changed significantly after sterilization (Beringer et al., 2015). Therefore, the selection of a sterilization method for piezoelectric coatings on titanium surfaces should balance sterility requirements with the stability of the functional interface. Coating structure, surface properties, and piezoelectric performance should all be assessed after sterilization.

Table 2 summarizes the main steps, advantages, and limitations of the major fabrication methods for piezoelectric coatings. Overall, no single fabrication route is optimal for all titanium-based piezoelectric coatings. Selection should reflect material type, substrate structure, implantation site, and intended function. Future strategies should jointly control phase, polarization, thickness, porosity, morphology, and interfacial bonding. Quantitative relationships among processing, wet-state output, long-term stability, and biological effects are also needed.

**TABLE 2:** Major fabrication methods and processing considerations for piezoelectric coatings on implant surfaces.

Method	Main steps	Key advantages	Key limitations
Hydrothermal synthesis and *in situ* conversion	Formation of TiO_2_ nanotubes or a porous oxide layer, hydrothermal reaction with metal-ion precursors, and optional annealing and poling	*In situ* growth can provide good interfacial bonding while preserving nano- or porous structures; equipment requirements are relatively low	The process is slow and parameter-sensitive; uniform coating of complex pore walls and batch-to-batch reproducibility remain challenging
Electrospinning	Preparation of a polymer or particle-containing solution, electrospinning onto Ti or PDA-modified Ti, and optional annealing and poling	A mild and relatively low-cost route to biomimetic fibrous interfaces; electric-field drawing can promote polymer-chain orientation	Coverage of complex three-dimensional pore interiors is limited; adhesion, wear resistance, and batch consistency require further optimization
Physical thin-film deposition	Substrate cleaning and pretreatment, vacuum deposition, and post-deposition annealing or poling as needed	Provides precise compositional and thickness control, with uniform, dense films and good reproducibility on planar surfaces	High equipment and operating costs; line-of-sight limitations, residual stress, and thermal mismatch constrain use on complex porous implants
Electrophoretic deposition	Preparation of a stable particle suspension, electric-field-driven deposition on conductive Ti, drying and sintering/heat treatment, and optional poling	Simple and relatively low-cost; readily tunes composite composition and is applicable to conductive substrates with complex external shapes	Uniformity depends on suspension stability and field distribution; drying or sintering can cause cracking, residual stress, and reduced adhesion
Layer-by-layer assembly	Alternating adsorption, rinsing, and drying of oppositely charged polyelectrolytes, nanoparticles, antimicrobial agents, or drugs	A low-temperature, highly controllable route for secondary functionalization of an existing piezoelectric layer	Repeated deposition cycles are time-consuming; interlayer stability and scalability remain insufficiently demonstrated
Poling treatment	Application of a contact or corona electric field, often with heating, to align ferroelectric domains or polymer dipoles, followed by cooling and performance testing	Directly enhances piezoelectric response and surface charge without changing the bulk coating structure	Uniform poling of complex three-dimensional structures is difficult, and polarization may decay during storage or service

## 5 Biological effects and mechanisms of piezoelectric coatings on titanium surfaces

As discussed in Section 4, the performance of titanium-based piezoelectric coatings is governed not only by material composition but also by processing-dependent structural, electrical, and interfacial characteristics. These properties help explain why coatings based on the same material can produce different biological effects. Poling controls the alignment of ferroelectric domains or molecular dipoles and affects the magnitude and stability of the piezoelectric response (Jin et al., 2021). Crystallinity, crystal phase, and preferred orientation determine the proportion and spatial arrangement of the electroactive phase (Yin et al., 2022). Coating thickness introduces a trade-off. Thin coatings can be strongly affected by substrate clamping, interfacial dead layers, and depolarization fields. Excessive thickness, however, may increase residual stress and the risk of cracking or delamination (Zhao et al., 2002; Nguyen et al., 2013; Kelley et al., 2020). Porosity and surface microstructure, including grain size, fiber orientation, and nanorod morphology, affect local strain distribution, charge generation, and the surface area available for interactions with ions, proteins, and cells. Porous or nanostructured coatings may provide favorable sites for cell attachment and mineral deposition, but excessive porosity may disrupt coating continuity and reduce mechanical load transfer. Interfacial bonding is also critical because it governs strain transfer from the substrate to the piezoelectric phase and coating stability under wet cyclic loading.

Because the coating forms the immediate interface between the implant and body fluids, proteins, and cells, these structural and interfacial characteristics may also affect wettability, protein adsorption, cell adhesion, and bacterial colonization independently of piezoelectricity. Other factors, including material composition, degradation products, ion release, and poling-induced static charge, may also contribute to the observed responses. Most existing studies cannot fully separate these factors from the effects of dynamic piezoelectric stimulation. Reported osteogenic, antibacterial, and immunomodulatory outcomes should therefore be interpreted as combined effects rather than attributed solely to piezoelectricity. Table 3 summarizes the biological models, reported effects, and major limitations of representative titanium-based piezoelectric coatings. The following subsections discuss these biological findings in relation to material composition, coating structure, electrical state, and interfacial properties.

**TABLE 3:** Biological evidence and key limitations of representative piezoelectric coatings on titanium surfaces.

Material type	Biological model	Biological effect	Key limitation	Ref.
Inorganic piezoceramic: BaTiO_3_	BMSCs; rabbit radial segmental defect	Enhanced cell adhesion and spreading, osteogenic-marker expression, bone ingrowth, osseointegration, and pull-out force under LIPUS	LIPUS itself is bioactive; electrical output, wet-state retention, and long-term mechanical durability were not quantified	(Fan et al., 2020)
Lead-free piezoceramic: BST	SBF mineralization assay; osteoblasts	Enhanced calcium-phosphate deposition, osteoblast adhesion, and proliferation after polarization	No animal validation; Sr incorporation, rod morphology, and residual charge are confounded with piezoelectricity	(Wu et al., 2024b)
Lead-free piezoceramic: BCZT/TiO_2_	SBF mineralization assay; osteoblasts	Promoted apatite deposition, osteoblast adhesion, and proliferation under loading or LIPUS	No animal validation; nanotopography, ceramic composition, residual charge, and dynamic piezoelectricity were not independently separated	(Wu et al., 2023a)
Polymer–ceramic composite: PLLA/HA	MG-63 cells; SBF mineralization assay	Improved apatite-forming ability and osteoblast-related cell response; PDA improved coating adhesion	No animal evidence; the effects of HA content, fiber morphology, PDA, and piezoelectricity cannot be separated	(Fotoukian et al., 2024)
Piezoelectric hydrogel composite: PWH/GelMA/SA	BMSCs; rat femoral defect model	Promoted cell spreading, osteogenic gene/protein expression, mineralization, and bone formation under LIPUS	Short observation period; long-term hydrogel degradation, wear resistance, and electrical stability remain unresolved	(Wang et al., 2026)
Inorganic piezoceramic: BaTiO_3_ on TPMS scaffold	L929 cells and rBMSCs; rabbit femoral condyle defect	Enhanced cell adhesion, migration, invasion, and osteogenic differentiation through focal-adhesion and PI3K/AKT-related pathways	The contributions of TPMS topology, load distribution, surface chemistry, and piezoelectricity remain coupled; long-term durability was not evaluated	(Wu et al., 2025)
Drug-loaded piezoceramic: TiO_2_@BaTiO_3_–vancomycin	*Staphylococcus aureus*; rat osteoblasts; rat subcutaneous implantation	Reduced early vancomycin release, prolonged antibacterial activity, and maintained osteoblast compatibility	No bone infection or osseointegration model; the key effect is static charge-regulated drug retention rather than directly demonstrated dynamic piezoelectricity	(Wu et al., 2021c)
Antibacterial piezoceramic composite: BST-Ag	Bacterial assays, osteoblasts, and SBF mineralization assay	Reduced bacterial adhesion and improved antibacterial activity by combining Ag^+^ retention with residual surface charge; supported osteoblast growth	No animal validation; Ag^+^ release, surface charge, rod geometry, and piezoelectricity are coupled	(Wu et al., 2024a)
Multilayer piezoelectric polymer system: 3DMA (TiO_2_/PVDF)	*Staphylococcus aureus*, *Escherichia coli*, RAW264.7 macrophages, and osteogenic cells; rat distal femoral models	Reduced bacterial adhesion and biofilm formation, promoted an anti-inflammatory macrophage phenotype, and supported osteogenesis	PVDF piezoelectricity, charge trapping, nanotopography, and static surface-charge effects were not independently resolved; long-term durability is unknown	(Sun et al., 2023)
Piezoelectric polymer: PVDF on laser-textured Ti6Al4V	*In vitro* bacterial and osteoblast-related assays	Reduced bacterial viability and early adhesion; laser texture and PVDF hydrophobicity contributed to the response	No direct quantitative piezoelectric output or *in vivo* evidence; the contributions of laser texture, hydrophobicity, and electroactivity remain inseparable	(Silva et al., 2026)
Oxygen-deficient piezoceramic hybrid: BaTiO_3_-x/L-arginine	MRSA, BMSCs, and macrophages; rat tibial infection model	Ultrasound-triggered ROS–NO–ONOO^-^ radical-chain antibacterial activity, followed by stage-dependent immune regulation and osteogenesis	Oxygen vacancies, L-arginine, radical chemistry, ultrasound, and piezoelectricity are co-mechanisms; long-term electrical and coating stability are unknown	(Sun et al., 2024)
Piezoceramic–nanozyme heterostructure: BaTiO_3_/Mn-MIL-100	Bacterial assays, macrophage and fibroblast-related assays; rat subcutaneous infection model	Local electric-field-enhanced membrane electroporation and ROS-mediated killing, followed by oxidative-stress reduction and repair-oriented immune regulation	No osseous infection or bone-integration model; nanozyme activity, heterojunction effects, tip geometry, and piezoelectricity are inseparable	(Ding et al., 2026)
Inorganic piezoceramic: BaTiO_3_ on porous Ti6Al4V	RAW264.7 macrophages and osteoblast-related cells; rat subcutaneous model; sheep cervical corpectomy model	Promoted M2-like macrophage polarization, suppressed MAPK/JNK signaling, enhanced oxidative phosphorylation, and supported bone repair under LIPUS	The biological contribution of LIPUS cannot be completely separated; long-term output retention, wear, and fatigue resistance were not evaluated	(Wu et al., 2023b)
Ferroelectric piezoceramic: ferroTi (BaTiO_3_)	BMDMs, BMSCs, and MRSA; rat femoral osteomyelitis model; subcutaneous biosafety model	Sequential dynamic antibacterial and static osteogenic immunomodulation: enhanced macrophage phagocytosis during infection and reparative polarization after infection control	Rodent evidence only; the contribution of static ferroelectric potential versus ultrasound-induced dynamic signaling requires further long-term validation	(Li et al., 2026)

### 5.1 Promotion of osteogenic differentiation and osseointegration

Conventional coatings mainly depend on material composition or surface roughness. In contrast, piezoelectric coatings can convert mechanical stimulation into dynamic electrical signals. Their central mechanism is the formation of an electrical microenvironment at the titanium-bone interface that is similar to that of mechanically loaded natural bone. This microenvironment can influence cell behavior through effects on the cell membrane (Figure 2).

**FIGURE 2:**
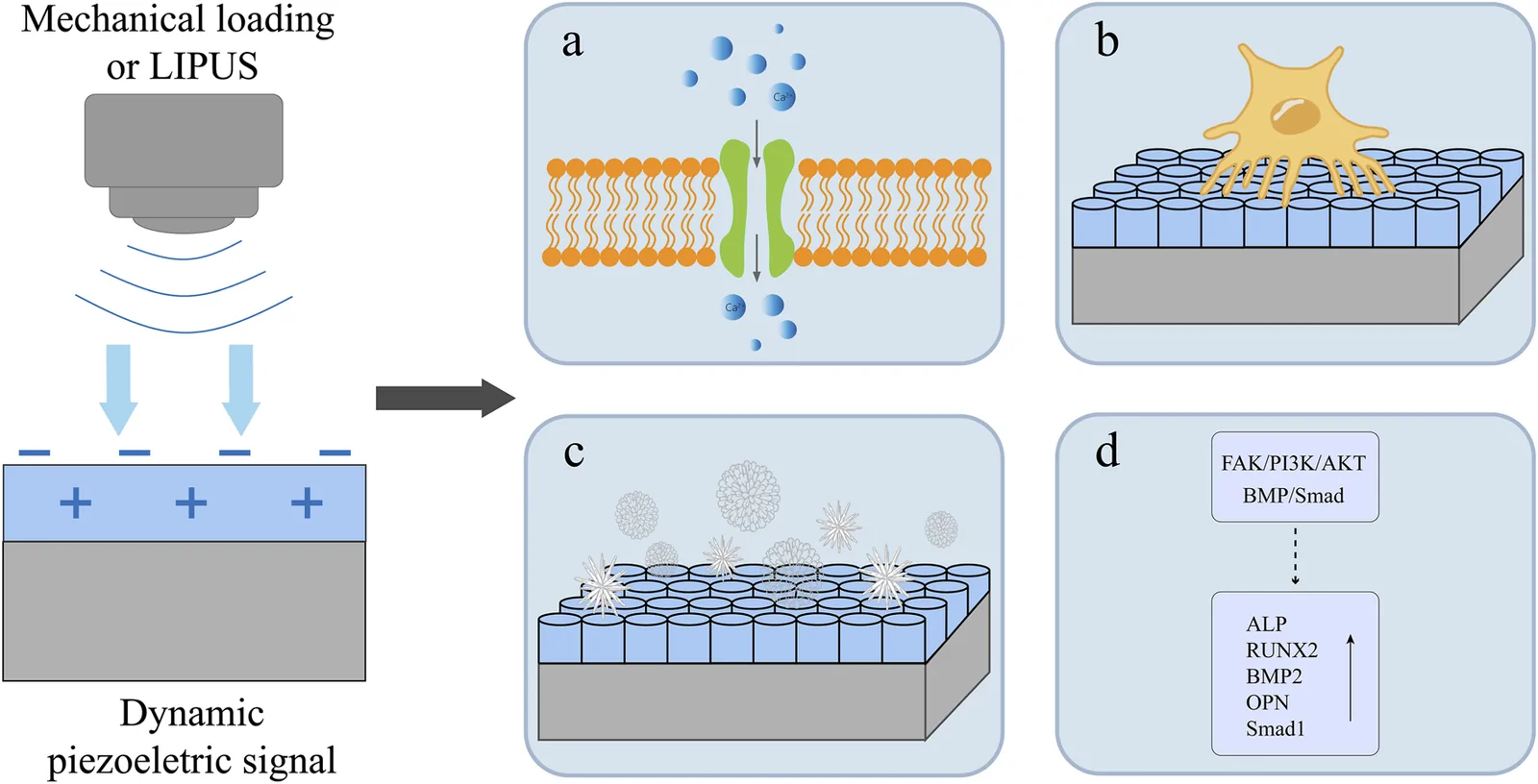
Schematic illustration of the osteogenic mechanisms induced by piezoelectric coatings on titanium-based implants. **(a)** activation of cell membrane ion channels and promotion of Ca^2+^ influx; **(b)** enhancement of osteogenic cell adhesion, spreading, and focal adhesion formation; **(c)** promotion of extracellular matrix mineralization and hydroxyapatite deposition; **(d)** activation of osteogenesis-related signaling pathways and upregulation of osteogenic marker expression.

Among inorganic piezoelectric ceramics, BaTiO_3_ is the most representative material in bone implant research (Khare et al., 2020). Fan et al. coated porous Ti-6Al-4V scaffolds (pTi) with BaTiO_3_ and combined them with LIPUS for the repair of large bone defects. Bone marrow mesenchymal stem cells (BMSCs) in the BaTiO_3_/pTi plus LIPUS group showed better adhesion and spreading. Expression of filamentous actin (F-actin) and vinculin increased, indicating improved cytoskeletal tension and focal adhesion maturation. Osteogenic markers including alkaline phosphatase (ALP), collagen type I (COL-I), runt-related transcription factor 2 (RUNX2), and osteopontin (OPN) were also upregulated. *In vivo*, the treatment promoted new bone ingrowth into scaffold pores, strengthened the bone-implant interface, and increased pull-out force (Fan et al., 2020). Doping with bioactive elements is another effective way to improve implant bioactivity. Strontium is a divalent trace element with bone affinity. Its chemical properties and biological transport pathways are similar to those of Ca^2+^, and it is closely related to bone metabolism (Pors Nielsen, 2004). Wu et al. added Sr to a BaTiO_3_ coating and formed a barium strontium titanate rod array on titanium. Under force or ultrasound, the rods generated local positive and negative potentials. These potentials promoted Ca^2+^ and PO_4_^3-^ accumulation and hydroxyapatite nucleation. Sr doping also provided osteogenic activity and may have lowered the threshold for effective charge generation under small loads (Wu et al., 2024b). Lead-free titanates such as BCZT further expand the available material systems. Wu et al. used TiO_2_ nanotubes as a transition layer to prepare a BCZT/TiO_2_ coating with a strong piezoelectric response. Remanent charge on the polarized surface promoted Ca^2+^ accumulation and bone-like apatite deposition in simulated body fluid. Simulated physiological loading or LIPUS supplied additional piezoelectric charge and further improved calcium phosphate deposition and osteoblast adhesion (Wu C. et al., 2023).

Piezoelectric polymers and their composite coatings provide flexible interfaces and structures similar to the extracellular matrix. Electrospun PLLA/HA fiber coatings can form a porous collagen-like network on titanium surfaces. HA provides osteoconductivity and sites for calcium phosphate nucleation. PLLA acts as a biodegradable piezoelectric framework and may generate local electrical stimulation under dynamic conditions (Fotoukian et al., 2024). TiO_2_@PVDF nanofiber membranes further show how surface potential affects osteogenic behavior. TiO_2_ nanoparticles can promote formation of the PVDF β phase and improve the piezoelectric response. A high surface potential can improve the adhesion of rat bone marrow mesenchymal stem cells (rBMSCs) and fibronectin secretion. It may also increase cell membrane permeability and promote the opening of voltage-gated Ca^2+^ channels (Liu et al., 2023). PVDF-ZnO nanofiber coatings combine improved piezoelectric output with the biological activity of Zn^2+^. Under simulated physiological strain, they promote F-actin stress fibers, focal adhesion formation, collagen deposition, and mineralization (Vaidya et al., 2025).

In titanium-based piezoelectric interfaces, hydrogels are better understood as flexible carriers or soft interfacial architectures than as a separate piezoelectric material class. They can carry piezoelectric particles, improve compliance, and regulate the local hydration environment. Recently developed piezoelectric hydrogel coatings combine a soft interface, electrical stimulation, and ion release in one system (Yao et al., 2025). A titanium-based dual-network coating combines a gelatin methacryloyl/sodium alginate (GelMA/SA) hydrogel with piezoelectric whitlockite (PWH) nanoparticles and is denoted Ti-GS/PWH. The GelMA/SA matrix provides a hydrated and soft three-dimensional environment that supports cell spreading. The PWH nanoparticles provide the piezoelectric response and continuously release Mg^2+^. LIPUS activates mechanical-to-electrical conversion. This system can upregulate osteogenesis-related genes, including RUNX2, bone morphogenetic protein 2 (BMP-2), OPN, and Smad1. It also increases the expression of BMP-2, COL-I, OPN, and osteocalcin (OCN) proteins. These findings suggest that it promotes BMSC differentiation through the bone morphogenetic protein (BMP)/Smad signaling pathway, collagen matrix formation, and local electrical stimulation (Wang et al., 2026). Compared with hard ceramic coatings, these hydrogels place more emphasis on the combined regulation of electrical signals, ion release, and a soft matrix environment.

The macroscopic scaffold structure also affects the transfer of piezoelectric signals to the cellular interface. Wu et al. prepared a gyroid-sheet triply periodic minimal surface (TPMS) porous titanium scaffold. Its smooth curved structure reduced stress concentration and transferred mechanical loads more evenly to the scaffold surface. A BaTiO_3_ coating then converted these mechanical signals into electrical stimulation. The coated TPMS scaffold increased vinculin-related adhesion signaling and activated the focal adhesion kinase (FAK)/phosphoinositide 3-kinase (PI3K)/protein kinase B (AKT) pathway. It therefore promoted the adhesion, migration, invasion, and osteogenic differentiation of rBMSCs (Wu et al., 2025). These results show that the osteogenic effect depends not only on the electrical properties of the coating. It also depends on the ability of the scaffold to transfer and distribute external mechanical stimulation.

Current mechanisms proposed for osteogenic differentiation and osseointegration are inferred mainly from *in vitro* proliferation, osteogenic markers, and mineralization endpoints. Direct evidence linking local piezoelectric signals to specific ion channels or intracellular pathways remains limited. Systematic validation at the *in vivo* tissue level is also insufficient. Differences in materials, stimulation conditions, and endpoints further prevent genuinely comparable quantitative conclusions across studies.

### 5.2 Antibacterial and anti-infective effects

Conventional antibacterial coatings usually depend on antibiotics or the release of antibacterial components such as Ag, Cu, and Zn. They can provide good short-term antibacterial effects. However, they may show burst release, weak later activity, possible cytotoxicity, and a risk of drug resistance (Chouirfa et al., 2019). Piezoelectric coatings can modulate the local interfacial microenvironment under mechanical stimulation. Surface charge, dynamic electrical potential, or ultrasound-activated piezocatalysis can affect drug release, bacterial adhesion, bacterial membrane integrity, and reactive oxygen species (ROS) and reactive nitrogen species (RNS) generation (Figure 3).

**FIGURE 3:**
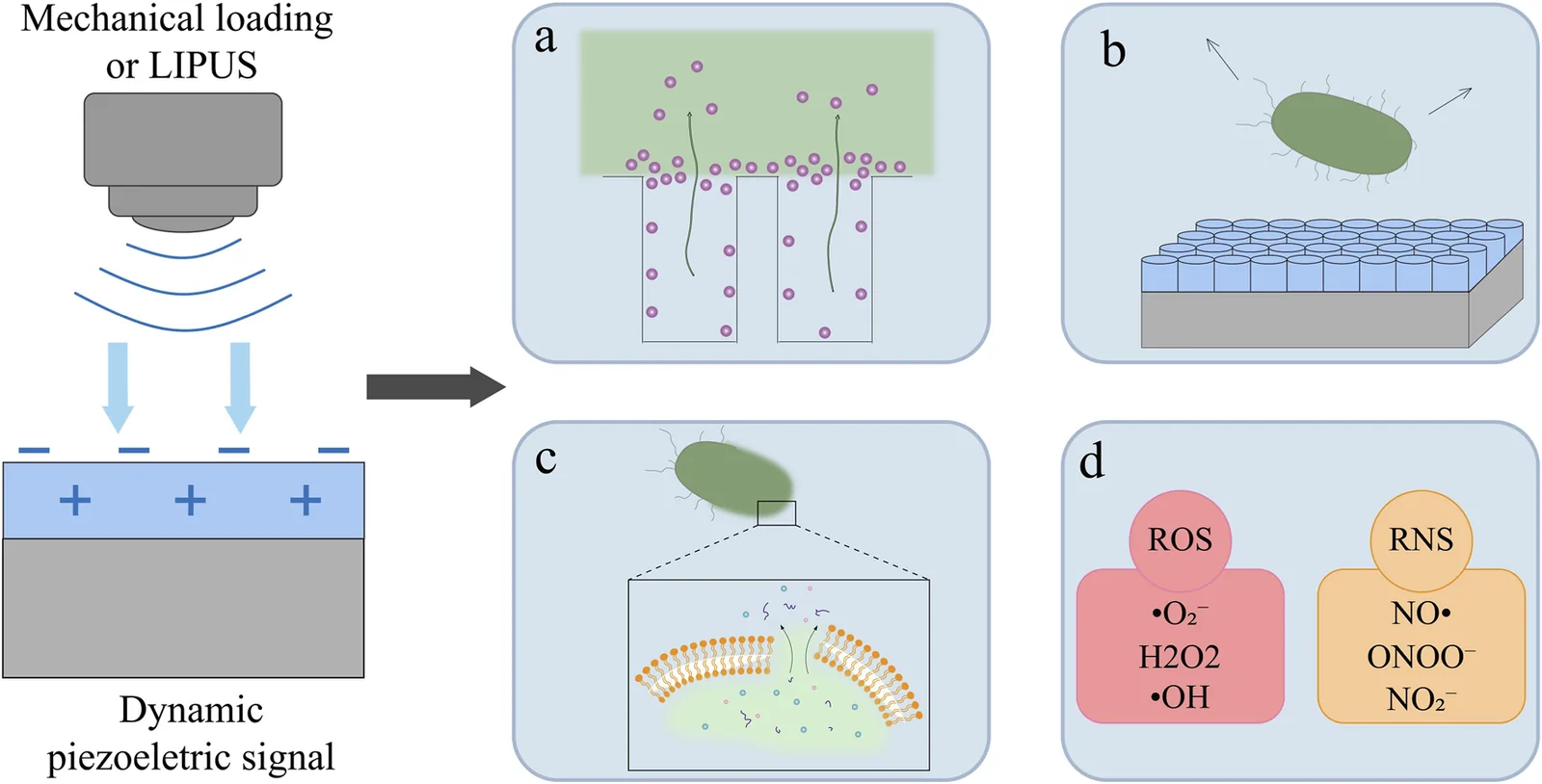
Schematic illustration of the antibacterial mechanisms of piezoelectric coatings on titanium-based implants: **(a)** regulation of the release of antibacterial agents or metal ions; **(b)** inhibition of early bacterial adhesion and biofilm formation; **(c)** disruption of bacterial membrane integrity and induction of intracellular component leakage; **(d)** ultrasound-activated piezoelectric effects that enhance ROS/RNS generation.

Release-control strategies use piezoelectric polarization charge to regulate the interfacial distribution and release pattern of antibacterial drugs or metal ions. Conventional TiO_2_ nanotubes have potential for local drug delivery on titanium surfaces, but they often show early burst release. Wu et al. prepared vancomycin-loaded TiO_2_@BaTiO_3_ coaxial nanotube coatings. After BaTiO_3_ was introduced, polarization left remanent charge on the nanotube surface. Charged vancomycin was retained near the nanotube openings, creating a dam-like effect. Therefore, drug release changed from simple diffusion along a concentration gradient to a two-stage process. The drug moved from the nanotube interior to a surface retention region and then to the external environment. This process reduced the diffusion rate, maintained a high local drug concentration, and provided lasting antibacterial activity (Wu et al., 2021c). Similarly, a BST-Ag rod array coating used residual negative charge on polarized BST to adsorb Ag^+^. This created a local antibacterial region with a high Ag^+^ concentration on the coating surface (Wu et al., 2024a). These strategies do not simply increase total Ag^+^ release. Instead, they increase the retention time and local accumulation of antibacterial agents on the implant surface. Therefore, their main function is not direct bacterial killing by piezoelectric materials. They act as local antibacterial delivery platforms supported by piezoelectric charge.

Non-release electrical strategies focus on the direct effect of surface charge on bacterial membranes. The three-dimensional multifunctional architecture (3DMA) developed by Sun et al. contains lower TiO_2_ nanotubes and an upper PVDF piezoelectric nanofiber layer. The pore size and potential of the PVDF layer separate bacteria from macrophages. Larger macrophages remain on the nanofiber layer, while smaller bacteria can pass through it and attach to the lower TiO_2_ nanotube layer. Positive charge traps then damage the bacterial membrane. This design provides electrical antibacterial activity without antibiotics or metal ion release (Sun et al., 2023). In comparison, a PVDF coating on laser-textured Ti-6Al-4V has a milder effect. It mainly uses laser texture, PVDF hydrophobicity, and β phase electroactivity to reduce initial bacterial adhesion (Silva et al., 2026). These systems have limited bactericidal strength. However, they do not depend on antibiotics, metal ions, or highly oxidative radicals and therefore have a relatively wide safety range. They may be suitable for long-term surfaces with a low risk of bacterial adhesion.

Piezoelectric sonodynamic antibacterial coatings provide a more active infection-control strategy. A BaTiO_3_-x/L-arginine (LA) coating uses oxygen vacancy engineering to improve the catalytic efficiency of BaTiO_3_ nanorod arrays under ultrasound. Ultrasound causes BaTiO_3_-x to generate a piezoelectric field and promotes electron-hole separation. Oxygen vacancies further capture electrons and improve O_2_ adsorption, increasing the generation of hydroxyl and superoxide radicals. Grafted LA releases nitric oxide (NO) in response to ROS. NO then reacts with superoxide radicals to form strongly oxidative peroxynitrite (ONOO^−^). This creates an ROS, NO, and ONOO^−^ radical chain reaction. The reaction damages the membranes of methicillin-resistant *Staphylococcus aureus* (MRSA), increases membrane permeability, and further disrupts deoxyribonucleic acid (DNA) and energy metabolism (Sun et al., 2024). This study moved piezoelectric sonodynamic antibacterial treatment beyond simple ROS generation. It introduced a radical chain reaction that also works with the immune response to kill bacteria.

Strategies based on local electric field enhancement further expand piezoelectric antibacterial mechanisms. Ding et al. developed a composite piezoelectric nanozyme containing manganese-based metal-organic framework MIL-100 (Mn-MIL-100) and BaTiO_3_. Under ultrasound, the interface between BaTiO_3_ and Mn-MIL-100 promoted charge separation. Positive charge accumulated at the highly curved tips of Mn-MIL-100 and formed a strong local electric field. This field disturbed the transmembrane potential of bacteria and caused membrane electroporation. Membrane permeability increased and intracellular substances leaked out. Local ROS further increased oxidative damage to the membrane (Ding et al., 2026). Compared with conventional ROS-based killing, this mechanism focuses on spatial concentration of piezoelectric charge and direct physical damage by the local electric field. Mn-MIL-100 also has nanozyme activity. After infection control, it can remove excessive ROS, reduce local oxidative stress, and support a repair environment. Therefore, this design follows a staged approach that first controls bacteria and then promotes repair.

Overall, the antibacterial activity of piezoelectric coatings is typically determined by multiple interacting factors. Dynamic piezoelectric signals, together with the associated surface charges and reactive oxygen species generation, may contribute to the regulation of bacterial adhesion and survival. Metal-ion release, material composition, surface topography, and static polarization charges can also exert antibacterial effects. The antibacterial outcomes reported to date should therefore not be attributed solely to piezoelectricity. Future studies should incorporate non-piezoelectric controls and strategies that suppress ion release or reactive oxygen species generation to distinguish the relative contributions of these factors.

### 5.3 Immune regulation and the osteoimmune microenvironment

The immune effects of piezoelectric coatings connect their osteogenic and antibacterial functions. After implantation, macrophages usually reach the interface before osteoblasts and help determine the local inflammatory environment. Macrophage phenotypes are often described as pro-inflammatory M1-like and reparative M2-like states. M1-like macrophages help remove pathogens and necrotic tissue. However, continuous M1-like activation can cause chronic inflammation, fibrous encapsulation, and poor osseointegration. M2-like macrophages secrete factors such as interleukin-10 (IL-10), transforming growth factor-beta (TGF-β), and bone morphogenetic protein-2 (BMP-2), which support inflammation resolution, vascularization, and bone formation. It should be noted that M1-like and M2-like phenotypes do not represent discrete macrophage states *in vivo*, but rather constitute a simplified experimental framework for describing distinct functional tendencies. Macrophages at the implant interface are highly heterogeneous and plastic, and their phenotypes continuously evolve in response to local infection, mechanical cues, and the progression of tissue repair. Therefore, the immune value of piezoelectric materials should not be described only as reducing inflammation or promoting M2-like polarization. Local electrical signals, surface charge, and metabolic changes can regulate macrophage functions at different stages. This regulation may create an osteoimmune microenvironment that supports osseointegration.

In a non-infected or mildly inflamed environment, piezoelectric electrical stimulation mainly promotes macrophage conversion toward an anti-inflammatory and reparative phenotype. Under dynamic loading or LIPUS, a BaTiO_3_-coated Ti-6Al-4V scaffold developed by Wu et al. promoted M2-like polarization. It also reduced M1-like and pro-inflammatory markers such as inducible nitric oxide synthase (iNOS) and tumor necrosis factor-alpha (TNF-α). More importantly, electrical stimulation inhibited the mitogen-activated protein kinase (MAPK)/c-Jun N-terminal kinase (JNK) inflammatory cascade. It also increased mitochondrial electron transport, oxidative phosphorylation, and adenosine triphosphate (ATP) production. Macrophage metabolism therefore shifted from a mainly glycolytic pro-inflammatory state toward an oxidative and reparative state (Wu H. et al., 2023). These findings suggest that piezoelectric coatings may promote M2-like polarization through immune metabolic changes, rather than only changing surface markers. This response may indirectly support bone repair. In dental implants, an occlusion-activated BCZT implant further showed that physiological loading can trigger immune regulation. Polarized BCZT generated controllable electrical signals under occlusal pressure. In RAW 264.7 macrophages, these signals reduced M1-like and pro-inflammatory markers, including cluster of differentiation 86 (CD86), TNF-α, interleukin-12 (IL-12), and interleukin-6 (IL-6). They increased M2-like and anti-inflammatory markers, including cluster of differentiation 206 (CD206), TGF-β, arginase-1 (Arg-1), IL-10, and interleukin-13 (IL-13). Transcriptomic results further suggested downregulation of hypoxia-inducible factor-1 (HIF-1)-related glycolytic genes and inhibition of nuclear factor kappa B (NF-κB), MAPK, and Janus kinase/signal transducer and activator of transcription (JAK/STAT) inflammatory pathways (Chen et al., 2026). The resulting environment favored anti-inflammatory repair. Future coating designs may therefore make greater use of mastication, walking, and joint movement. They should not depend only on external ultrasound or *in vitro* dynamic loading.

In an infected implant environment, immune regulation should not always aim to maintain M2-like polarization. Moderate M1-like activation during early infection can improve macrophage phagocytosis and bacterial clearance. Premature inhibition of M1-like activity may instead weaken antibacterial defense. The BaTiO_3_-x/LA coating follows this stage-dependent immune strategy. Under ultrasound, ROS causes L-arginine to release NO. NO takes part in the ROS, NO, and ONOO^−^ antibacterial chain reaction. It also promotes macrophage conversion toward an iNOS-positive M1-like phenotype and strengthens early immune killing. After infection is controlled and L-arginine is consumed, the BaTiO_3_-x/LA nanorods promote a shift toward a CD206-positive M2-like phenotype. This supports inflammation resolution and osseointegration (Sun et al., 2024). The ferroTi system developed by Li et al. further defined this staged regulation as sequential bioelectrical programming. Ferroelectric BaTiO_3_ nanostructures were grown *in situ* on titanium. The system provided both ultrasound-activated dynamic electrical signals and a static signal from the inherent potential of the ferroelectric surface. During early infection, the dynamic signal activated voltage-gated Ca^2+^ channels and promoted Ca^2+^ entry. It increased M1-like and phagocytosis-related signals such as iNOS, TNF-α, and Fc gamma receptor (FcγR). This response improved macrophage phagocytosis and clearance of MRSA. After infection control, the static surface potential strengthened macrophage focal adhesions and extracellular matrix receptor interactions. It promoted an anti-inflammatory reparative phenotype. Macrophage-conditioned medium then increased ALP, OPN, OCN, and RUNX2 expression in BMSCs (Li et al., 2026). Switching between dynamic and static signals allowed macrophages to perform different tasks during infection and repair. This system provides a clear model for stage-dependent immune regulation at piezoelectric and ferroelectric interfaces. However, the strategy still relies on predefined ultrasound activation and signal switching. It cannot adjust signal intensity, frequency, or duration in real time according to the post-implantation inflammatory state. Future work should quantify relationships between local immune states and electrical parameters and develop temporally tunable interfaces aligned with host immune progression.

The Mn-MIL-100/BaTiO_3_ composite piezoelectric nanozyme developed by Ding et al. adds another immune pathway based on repair after bacterial killing. Under ultrasound, the system combines a strong local electric field with ROS to kill bacteria. After infection control, redox cycling between Mn^2+^ and Mn^3+^ provides nanozyme activity. This activity removes excessive ROS, reduces oxidative stress in macrophages, and promotes a shift from the M1-like to the M2-like phenotype (Ding et al., 2026). Strong antibacterial strategies often cause oxidative stress and inflammatory risk. Therefore, a piezoelectric anti-infective interface should not focus only on rapid bacterial killing. It should also reduce inflammation at the proper time and start tissue repair.

The figure shows the main mechanisms by which piezoelectric surface modification of titanium regulates the immune microenvironment (Figure 4). Immune regulation by piezoelectric coatings should therefore not be described simply as M2-like promotion or anti-inflammatory activity. In non-infected or mildly inflamed conditions, M2-like polarization supports osseointegration. During early infection, moderate M1-like activation can improve pathogen clearance. An ideal piezoelectric immune-regulating interface should adapt to the biological condition and change over time. It should strengthen host antibacterial immunity at the early stage. At later stages, it should reduce excessive inflammation and promote tissue repair. Stable osseointegration may then be achieved through an improved osteoimmune microenvironment.

**FIGURE 4:**
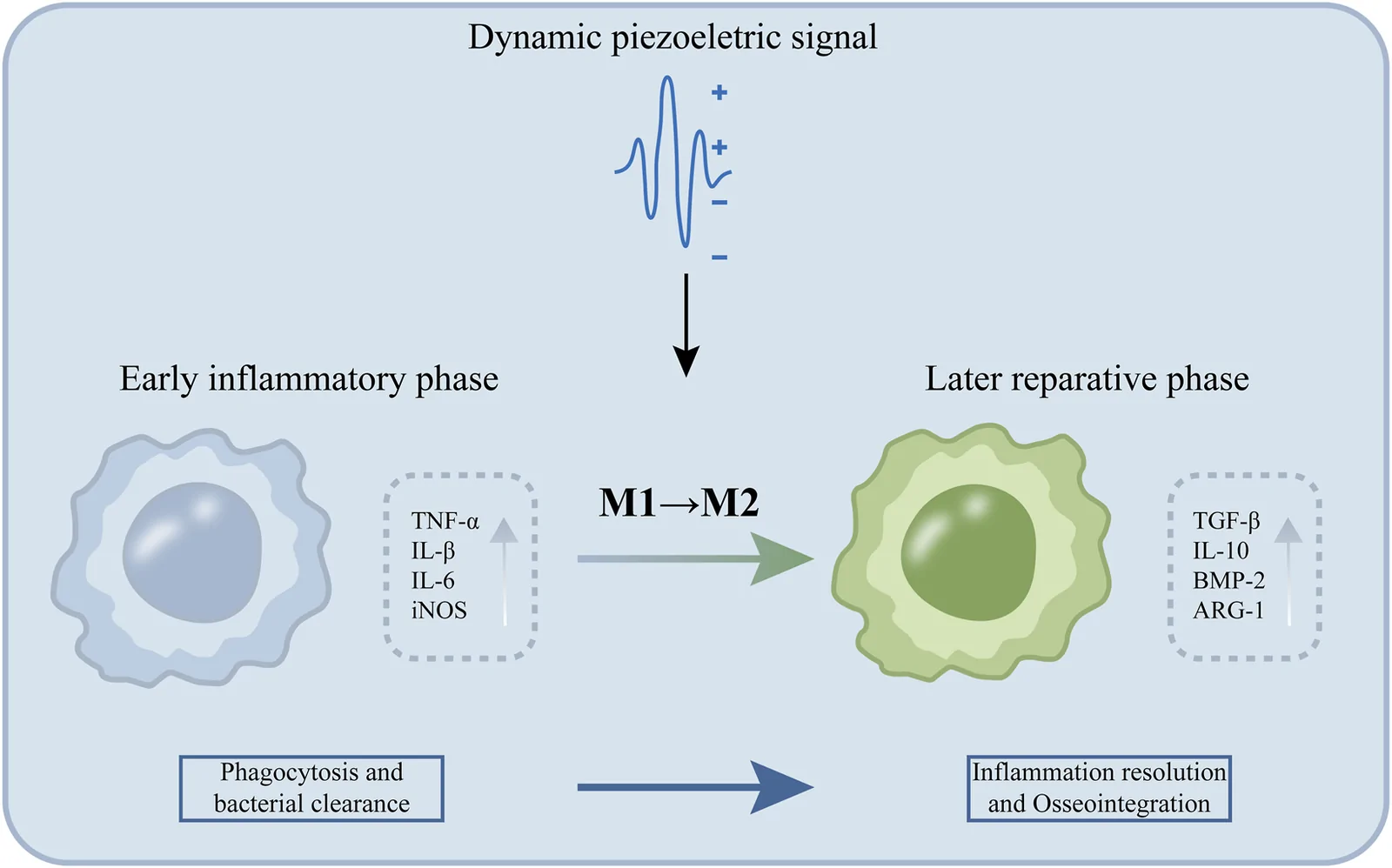
Stage-specific modulation of macrophage polarization.

## 6 Challenges and future perspectives

Piezoelectric coatings on titanium surfaces show strong potential for promoting osteogenic differentiation, controlling infection, and regulating the immune microenvironment. However, several challenges limit their further use. Table 4 summarizes the major translational barriers, recommended measures, and expected goals for titanium-based piezoelectric coatings.

**TABLE 4:** Translational roadmap and key tasks for piezoelectric coatings on titanium surfaces.

Translational stage	Current key barrier	Recommended measures and core metrics	Expected translational goal
Application and safety	Long-term Pb exposure, ion/particle release, and the wet-state stability of natural materials remain insufficiently characterized	Select materials according to implantation site and service time; evaluate release kinetics and long-term safety. Lead-containing materials may be prioritized for short-term or retrievable devices	Define application scenarios and acceptable safety boundaries
Wet-state electrical output	Dry-state parameters may not reflect actual wet-state output, and no unified signal-dose definition is available	Perform wet-state dynamic/*in situ* characterization; consistently report loading, frequency, output, and coating state to establish input-signal-effect relationships	Define biologically relevant signal-dose ranges
Interfacial durability	Micromotion and cyclic loading can cause cracking, delamination, wear, and output decay	Under wet cyclic loading, jointly assess adhesion, wear, fatigue resistance, polarization retention, and dynamic output	Maintain structural and functional reliability throughout service
Fabrication and quality control	The process is sensitive and batch variation is substantial; sterilization may alter coating structure and interfaces	Standardize fabrication and quality control; report batch variation in thickness, phase, morphology, adhesion, and wet-state output, and compare post-sterilization retention	Establish reproducible, sterilization-compatible fabrication
Mechanistic evidence	Piezoelectric signals are coupled with topography, material chemistry, ion release, and static polarization effects	Use rigorous controls, pathway inhibition, and time-resolved analysis to distinguish independent and synergistic effects	Establish attributable and verifiable mechanisms
Preclinical validation	Current evidence is mainly from short-term *in vitro* and small-animal studies and does not capture real clinical settings	Evaluate osseointegration, antibacterial activity, immunomodulation, and functional retention in large-animal, long-term, and disease models	Improve the clinical generalizability of experimental findings
Design standards and translation	No unified thresholds exist for electrical output, frequency, coating thickness, or adhesion strength	Develop comparable wet-state electrical, mechanical, and biological evaluation systems and define application-specific performance windows	Provide quantitative guidance for product design, scale-up, and translation

### 6.1 Wet-state electrical output

The effectiveness and stability of piezoelectric performance *in vivo* remain unclear. Current studies often use d_33_, piezoresponse force microscopy (PFM) response, or open-circuit voltage to evaluate coatings. However, ions, hydration layers, and protein adsorption in body fluids can screen surface charge. Measurements obtained under dry or ideal loading conditions may not represent the signals that cells sense during long-term cyclic loading *in vivo*. In recent years, invasive electrodes and implantable sensors have provided technical approaches for detecting electrical signals *in vivo*. For example, Kirschner wires or stainless-steel needle electrodes can be placed in direct contact with, or inserted into, bone tissue to record local bone potentials intraoperatively (Zigman et al., 2013; Ziran and Attias, 2024). Miniaturized implantable piezoelectric sensors can also enable *in situ* measurement of microvolt-scale piezoelectric signals under dynamic stimulation (Zhao et al., 2019). However, current measurements remain susceptible to electrode–tissue interface effects, motion artifacts, environmental noise, and interference from endogenous bioelectrical signals. Long-term, *in situ*, quantitative monitoring of electrical signals at the bone–implant interface also remains difficult. Future studies should place greater emphasis on surface potentials and dynamic electrical output under wet conditions, as well as on the time-dependent attenuation of these signals. *In situ* detection technologies suitable for the bone–implant interface should also be developed to further establish the relationships among electrical signal intensity, mode of activation, mechanical or acoustic input parameters, stimulation duration, and biological effects.

### 6.2 Interface durability

Interfacial bonding strength and long-term service stability remain important limits to clinical translation. Inorganic ceramic coatings have relatively strong piezoelectric responses and good chemical stability. However, they may be brittle and may develop residual stress, microcracks, or interfacial delamination. Polymer coatings such as PVDF and PLLA are more flexible. Their polarization decay, wear, performance after sterilization, and long-term adhesion still require evaluation. Hydrogel and composite coatings may also show swelling, softening, particle loss, or interfacial separation. Current evidence indicates that transition-layer construction, surface roughening, interfacial reaction control, and mechanical interlocking are the principal interface-engineering strategies that have been applied to titanium-based piezoelectric coatings. Their shared objective is to improve coating–substrate bonding and mechanical load transfer. Future studies should further enhance the structural reliability of these interfaces. In particular, uniform coating deposition on the inner walls of complex porous structures, fatigue- and delamination-resistant interface design, and the coordinated optimization of bonding strength and wet-state piezoelectric output remain insufficiently explored.

Tribological effects are another important translational limitation. Implant micromotion, orthopedic joint loading, and dental mastication can cause interfacial friction, coating wear, crack propagation, and particle detachment. Such damage may reduce coating integrity and piezoelectric output while altering surface chemistry and promoting particle or ion release. Long-term biosafety risks may consequently increase. Studies should jointly assess post-cycling adhesion, wear or delamination, polarization retention, and electrical output in relevant wet media.

### 6.3 Fabrication and quality control

Complex fabrication processes and non-standardized evaluation criteria continue to limit meaningful comparisons among studies, while batch-to-batch reproducibility remains a critical requirement for translation. Hydrothermal conditions, deposition parameters, coating thickness and microstructure, and subsequent poling can alter phase composition, crystallographic orientation, polarization state, and piezoelectric output. Multilayer composite coatings further involve surface pretreatment, deposition, drying, heat treatment, poling, and sterilization, making batch consistency and uniform coverage of complex geometries difficult to achieve. Therefore, standardized fabrication and quality-control systems should identify and control critical processing parameters while assessing thickness, phase composition, surface morphology, adhesion strength, d_33_, and dynamic electrical output. Reporting within-batch and between-batch variation, together with predefined acceptance criteria, would improve reproducibility, cross-study comparability, and the prospects for scale-up and clinical translation.

Sterilization may also affect the structural and functional stability of piezoelectric materials, with the extent of these effects depending on the material system and sterilization method. Previous studies have shown that electrospun PVDF-TrFE can retain stable piezoelectric output after combined ethanol and UV treatment (Beringer et al., 2015), whereas β-irradiation, although causing no significant changes in the crystalline phase or thermal behavior of P(VDF-TrFE-CFE), may alter certain dielectric properties (Della Schiava et al., 2020). Piezoelectric output, interfacial bonding, and biocompatibility should be compared systematically after different sterilization procedures.

### 6.4 Application and safety

The biosafety of materials and safe limits for ion release require systematic study. PZT can contain more than 60 wt% Pb, creating environmental and biological safety concerns for implant coatings. Barrier layers of polyimide, Parylene-C, polydimethylsiloxane (PDMS), or epoxy can isolate lead-containing layers from fluids and tissues. However, cracking, wear, delamination, or encapsulation failure may still release Pb. Encapsulation can reduce risk but cannot replace long-term *in vivo* safety testing. Given its high electromechanical performance, PZT may be more suitable for short-term, monitorable, and retrievable implantable sensors or microactuators (Wang and Dargusch, 2025). Lead-free ceramics and their composites are more suitable for biomedical use than lead-containing ceramics. However, long-term implantation may still cause particle loss, ion release, local accumulation, and possible toxicity. Functional ions such as Sr^2+^, Zn^2+^, Ag^+^, Cu^2+^, and Mg^2+^ can improve osteogenesis, antibacterial activity, or immune regulation. Excessive or rapid release may still cause damage. Therefore, multifunctional coatings should not simply contain more functional components. The safe concentration range, release behavior, and long-term tissue response of each component should be clearly defined.

Natural piezoelectric materials offer biocompatibility, degradability, and biomimetic properties, but their intrinsic responses are weak and strongly depend on molecular orientation, crystallinity, and assembly (Shin et al., 2020; Wang et al., 2020). Currently, direct studies using natural piezoelectric materials alone as the primary piezoelectric coating on titanium substrates remain limited; their structural and electromechanical-output stability under wet conditions, interfacial adhesion to titanium substrates, and long-term wear resistance warrant systematic evaluation. For complex titanium surfaces, the ability to form uniform coatings, retain performance after sterilization, and remain reliable under prolonged cyclic loading should also be assessed in future studies. Combining them with inorganic piezoelectric phases or stable polymers may balance biological function, electromechanical response, and long-term reliability.

### 6.5 Mechanistic evidence

The biological mechanisms of piezoelectric coatings also require further study. Many systems simultaneously change roughness, wettability, chemical composition, ion release, protein adsorption, and cell adhesion. It is therefore difficult to determine the actual contribution of the piezoelectric effect. Future studies should include depolarized controls and controls with the same morphology. Ion channel blocking, integrin or FAK inhibition, ROS removal, and signaling pathway inhibition can further define specific mechanisms. Transcriptomic, proteomic, metabolomic, and *in vivo* time-course analyses are also needed. These methods can clarify how piezoelectric signals affect osteogenic differentiation, bacterial membrane damage, radical generation, macrophage polarization, and immune metabolic changes. Most studies focus on osteoblasts, mesenchymal stem cells, and macrophages. Effects on endothelial cells, osteoclasts, and sensory neurons remain poorly understood. Angiogenesis, bone resorption, and sensory-neural regulation also contribute to bone repair and remodeling. Therefore, future studies should examine multiple cell types and their interactions to define the broader biological basis of osseointegration.

### 6.6 Preclinical validation

*In vivo* models and clinical translation studies also require further development. Current evidence mainly comes from *in vitro* cell experiments, small-animal bone defect models, or short-term antibacterial models. The clinical environment involves continuous loading, micromotion and wear, plaque or biofilms, immune responses, differences in bone quality, and underlying disease. Future studies should use more large-animal models and long-term implantation experiments. Relevant disease models include infected bone defects, osteoporosis, diabetes, and peri-implantitis. Coating methods should also be assessed for compatibility with current titanium implant manufacturing, sterilization, storage, and quality control.

Within this broader preclinical framework, evaluation should be tailored to the intended application. For dental implants, priority evaluation domains include coating retention under long-term multiaxial occlusal loading, performance in saliva and polymicrobial biofilms, peri-implant inflammation, marginal bone loss, and implant stability. For load-bearing orthopedic implants, priorities include fatigue and wear, particle or ion release, fixation strength, bone–implant contact, defect healing, infection recurrence, and functional recovery. Regulatory requirements may also differ depending on whether the coating acts as a passive surface modification, incorporates a drug or releasable ion, or requires external activation such as ultrasound. Because application-specific regulatory classifications and validated clinical endpoints have not yet been established for these coatings, defining them remains an important research need rather than part of an established translational pathway.

### 6.7 Design standards and translation

Importantly, current evidence does not support universal quantitative design criteria or performance thresholds for titanium-based piezoelectric coatings. Testing conditions vary substantially for piezoelectric coefficients, voltage or current output, stimulation frequency, loading mode, and biological endpoints. Dry-state measurements also cannot be directly extrapolated to wet physiological conditions under cyclic loading. Therefore, reported parameters and outcomes are not directly comparable and cannot define a universal optimal thickness, output, or stimulation dose. The absence of reproducible material–structure, wet-state-signal, and biological-effect relationships is itself a major knowledge gap. Future studies should consistently report thickness, polarization state, wet-state output, loading frequency and duration, adhesion, and long-term retention. Comparable evaluation systems can then support application-specific design standards.

Overall, the development of piezoelectric coatings on titanium surfaces should not focus only on increasing piezoelectric coefficients or adding functional components. Design should be guided by a local electrical microenvironment that can be activated, transferred, sensed, and maintained under realistic *in vivo* conditions. Progress in interfacial structure, wet-state signal measurement, long-term safety testing, standardized preparation, and clinically relevant models will support further development. Piezoelectric coatings may then become an important surface modification strategy for improving osseointegration, reducing infection risk, and regulating the osteoimmune microenvironment.

## 7 Conclusion

Piezoelectric materials provide a new approach to titanium implant surface modification that differs from conventional roughening, HA coatings, and antibacterial ion coatings. By converting physiological mechanical loads or external ultrasound into local electrical signals, piezoelectric coatings can reproduce the electrophysiological microenvironment of natural bone. They can also work with surface topography, mineralization, immune regulation, and antibacterial functions. At present, the reported quantitative values are descriptive rather than prescriptive, and universal thresholds for adhesion strength, wet-state electrical output, coating thickness, or stimulation dose cannot yet be defined. Current evidence still mainly comes from *in vitro* studies and small- or medium-sized animal models. Clinical translation requires further work on long-term piezoelectric stability, reliable interfacial bonding, *in vivo* signal dose, material safety, and standardized evaluation. Therefore, establishing standardized wet-state electrical, mechanical-stability, and biological evaluation systems is an important next step. Overall, piezoelectric coatings may help change titanium implants from passive biomaterials into smart bioactive interfaces. Such interfaces could sense the mechanical environment and regulate the local microenvironment.

## Glossary

BTO: barium titanate (BaTiO_3_)
BCZT: barium calcium zirconate titanate
BST: barium strontium titanate
ZnO: zinc oxide
PVDF: polyvinylidene fluoride
PLLA: poly-L-lactic acid
HA: hydroxyapatite
LIPUS: low-intensity pulsed ultrasound
PZT: lead zirconate titanate
P(VDF-TrFE): poly(vinylidene fluoride-co-trifluoroethylene)
AlN: aluminum nitride
Pr: remanent polarization
d_33_: longitudinal piezoelectric coefficient
CaTiO_3_: calcium titanate
CaO: calcium oxide
BMSCs: bone marrow mesenchymal stem cells
F-actin: filamentous actin
ALP: alkaline phosphatase
COL-I: collagen type I
RUNX2: runt-related transcription factor 2
OPN: osteopontin
PWH: piezoelectric whitlockite
BMP-2: bone morphogenetic protein 2
OCN: osteocalcin
TPMS: triply periodic minimal surface
FAK: focal adhesion kinase
PI3K: phosphoinositide 3-kinase
AKT: protein kinase B
rBMSCs: rat bone marrow mesenchymal stem cells
ROS: reactive oxygen species
RNS: reactive nitrogen species
3DMA: three-dimensional multifunctional architecture
LA: L-arginine
NO: nitric oxide
ONOO^−^: peroxynitrite
MRSA: methicillin-resistant *Staphylococcus aureus*
DNA: deoxyribonucleic acid
Mn-MIL-100: manganese-based metal-organic framework MIL-100
IL-10: interleukin-10
TGF-β: transforming growth factor-beta
iNOS: inducible nitric oxide synthase
TNF-α: tumor necrosis factor-alpha
MAPK: mitogen-activated protein kinase
JNK: c-Jun N-terminal kinase
ATP: adenosine triphosphate
CD86: cluster of differentiation 86
IL-12: interleukin-12
IL-6: interleukin-6
CD206: cluster of differentiation 206
Arg-1: arginase-1
IL-13: interleukin-13
HIF-1: hypoxia-inducible factor-1
NF-κB: nuclear factor kappa B
JAK/STAT: Janus kinase/signal transducer and activator of transcription
FcγR: Fc gamma receptor
PFM: piezoresponse force microscopy
EPD: electrophoretic deposition
LbL: layer-by-layer
d_31_: transverse piezoelectric coefficient
